# Genome-Wide Identification and Expression Profiling of Potato (*Solanum tuberosum* L.) Universal Stress Proteins Reveal Essential Roles in Mechanical Damage and Deoxynivalenol Stress

**DOI:** 10.3390/ijms25021341

**Published:** 2024-01-22

**Authors:** Tianshuai Qi, Fumeng He, Xinqi Zhang, Jiaqi Wang, Zengli Zhang, Heran Jiang, Biao Zhao, Chong Du, Yunzhu Che, Xu Feng, Yingnan Wang, Fenglan Li

**Affiliations:** 1 College of Life Sciences, Northeast Agricultural University, Harbin 150030, China; qtianshuai@163.com (T.Q.); hefumeng@neau.edu.cn (F.H.); zhangxinqi148@163.com (X.Z.); wjq13941677600@163.com (J.W.); zhangzenglia@outlook.com (Z.Z.); zhaob35@outlook.com (B.Z.); 18710381134@163.com (C.D.); 18246263290@163.com (Y.C.); 18045043687@163.com (X.F.); 2 College of Horticulture and Landscape Architecture, Northeast Agricultural University, Harbin 150030, China; heranjiang@163.com

**Keywords:** USP gene family, abiotic stress, bioinformatics analysis, transcriptome, DON

## Abstract

Universal stress proteins (USPs) play an important regulatory role in responses to abiotic stress. Most of the research related to USPs so far has been conducted on plant models such as *Arabidopsis* (*Arabidopsis thaliana)*, rice (*Oryza sativa* L.), and cotton (*Gossypium hirsutum* L.). The potato (*Solanum tuberosum* L.) is one of the four major food crops in the world. The potato is susceptible to mechanical damage and infection by pathogenic fungi during transport and storage. Deoxynivalenol (DON) released by *Fusarium* can seriously degrade the quality of potatoes. As a result, it is of great significance to study the expression pattern of the potato StUSP gene family under abiotic stress conditions. In this study, a total of 108 USP genes were identified from the genome of the Atlantic potato, divided into four subgroups. Based on their genetic structure, the physical and chemical properties of their proteins and other aspects of their biological characteristics are comprehensively analyzed. Collinear analysis showed that the homologous genes of StUSPs and four other representative species (*Solanum lycopersicum*, *Arabidopsis*, *Oryza sativa* L., and *Nicotiana attenuata*) were highly conserved. The cis-regulatory elements of the StUSPs promoter are involved in plant hormones, environmental stress, mechanical damage, and light response. RNA-seq analysis showed that there are differences in the expression patterns of members of each subgroup under different abiotic stresses. A Weighted Gene Coexpression Network Analysis (WGCNA) of the central gene showed that the differential coexpression gene is mainly involved in the plant–pathogen response process, plant hormone signal transduction, and the biosynthesis process of secondary metabolites. Through qRT-PCR analysis, it was confirmed that *StUSP13*, *StUSP14*, *StUSP15*, and *StUSP41* may be important candidate genes involved in the response to adversity stress in potatoes. The results of this study provide a basis for further research on the functional analysis of StUSPs in the response of potatoes to adversity stress.

## 1. Introduction

At present, the potato (*Solanum tuberosum* L.) is one of the four major grain crops in the world [1]. Because of its important commodity economic value and nutritional value, the potato is of great significance in ensuring world food security [2]. Potatoes are one of the main sources of carbohydrates and vitamins, which can provide a basic energy supply for human life activities. In addition, potatoes are rich in protein and in a variety of biologically active ingredients that are beneficial to human health, such as terpenoids, polysaccharides, alkaloids, and polyphenols [3,4,5]. The use of potatoes as raw materials for food processing and in the production of potato starch and snack products has driven the development of the potato industry [6,7]. However, most potato breeds are relatively susceptible to biological or abiotic stresses, such as pathogen infection throughout the plant’s entire growth and development in the field, mechanical damage to the tuber during picking and transportation, and extensive rot brought on by the entry of pathogenic bacteria, including fusarium, from the wound during storage as a result of inadequate management. Potato yield and quality will significantly decline as a result of the emergence of diseases, the aggravation of mechanical damage, and other risks [8,9]. Therefore, improving the ability of potatoes to resist adversity and stress is an important aim when attempting to improve potato breeds.

DON, the most prevalent B-type trichothecene compound, destroys normal cell physiological function, and indirectly damages mitochondria [10]. Fusarium head blight (FHB), caused by *Fusarium*, is the most important wheat disease in the world, significantly reducing crop yields [11]. *Fusarium* spp., particularly *Fusarium sambucinum*, *Fusarium graminearum*, *Fusarium avenaceum*, and *Fusarium oxysporum*, are the most notorious pathogenic fungi in various potato-growing regions, decreasing food quality by releasing DON [11,12]. In plants, strategies for responding to adversity include protecting cell membranes, removing reactive oxygen species (ROS), regulating the cell cycle, activating the antioxidant enzyme system, and producing defensive or protective proteins [11,13]. USP is an ancient and conserved protein that is found in bacteria, archaea, fungi, and plants [14]. USP was first discovered in the W3110K-12 strain of *E. coli* (*Escherichia coli*) and is a 12.5 kDa cytoplasmic protein [15]. In *E. coli*, the USP gene is divided into six groups [16]. USPs can be divided into two subgroups according to their ability to bind ATP. The uspA subgroup includes uspA, uspC, and uspD; the uspFG subgroup includes uspF and uspG [17]. In plants, genes with uspA domains similar to bacteria are defined as USP genes [18]. The physiological functions of USPs mainly include antioxidant stress [19], regulating cell growth and development [20], and other processes. In addition, USPs are also significantly induced under unfavorable environmental conditions, which plays a positive role in the cell’s tolerance to adversity [21]. Although many USPs have been characterized in terms of their structure and biochemistry, their role is still unknown.

Only the uspA domain is present in the *E. coli* USP structure, and uspA is involved in the reaction to hormones like salicylic acid, methyl jasmine, and abscisic acid [22]. However, the USPs of plants are functionally diversified to a large extent through additional auxiliary domains, allowing them to have many different functions [23]. Multiple USP genes have been studied in *Arabidopsis thaliana* [24], *Oryza sativa* L. [25], *Solanum lycopersicum* [26], *Gossypium herbaceum* L. [27], and other model plants. Existing studies have shown that USP genes can improve plant resistance and alleviate cell damage caused by various stresses [28]. *OsUSP1* from rice was the first USP gene discovered in the plant world. It is involved in the activation of the ethylene response signaling cascade during hypoxia and has been shown to have a positive regulatory effect on the flood resistance of rice [25]. It is reported that a total of 44 USP genes have been identified in *Arabidopsis* [29]. The *AtUSP* promoter can be induced by plant hormones and various stresses [30]. Among them, *AtUSP9*, *AtUSP12* [31], and *AtUSP17* have been identified as being involved in multiple stress responses. *AtUSP17* regulates the expression of *CCA1* and *TOC1*, the central genes related to circadian rhythm [20]. The *AtUSP17* gene was significantly induced by salt stress and injury [31], and the overexpression of *AtUSP17* enhanced the plant’s tolerance to oxidative stress and thermal stress [32,33]. *AtUSP17* regulates the salt resistance of *Arabidopsis* by regulating ethylene, abscisic acid, and reactive oxygen species [34]. In addition to *Arabidopsis*, in wild tomato (*Solanum pennellii* Correll.), the overexpression of *SpUSP* promotes the closure of pores to reduce the excessive harm that drought stress causes to individual plants, thereby enhancing drought resistance. On the other hand, oxidative stress, mechanical damage, and plant hormones can all induce the expression of *SpUSP* [35]. As a USP protein in tomatoes, *SlRd2* participates in the regulation of reactive oxygen species production and improves plant tolerance to salt and osmotic stress [26]. The overexpression of *GhUSP1* and *GhUSP2* in cotton can significantly improve drought tolerance [36], and *GhUSP2* can regulate the growth state of plants under high temperatures and salt stress [37]. USPs directly affect the growth and development of plants. The ectopic expression of *SbUSP* in *Nicotiana tabacum* L. can enhance salt tolerance [38], and the expression of *GhUSP1* positively regulates the accumulation of proline, soluble sugar, and chlorophyll in plants [27]. The USP gene is potentially involved in stress response mechanisms, including the accumulation of osmoregulatory substances, reducing the pore diameter by increasing the content of endogenous abscisic acid, enhancing antioxidant defense processes, promoting photosynthesis, maintaining the integrity of biofilms, and interacting with other general stress response proteins [35,39].

The role of USPs in model plants such as *Arabidopsis* has been fully studied. However, in many plants, the precise molecular function and regulatory response process of USPs are not yet clear. At present, there are no reports in the literature about the characterization of USPs in potatoes. The genome sequencing of the Atlantic potato has been completed, making it possible to analyze the whole genome of the potato USP gene family. In this study, the potato USP gene family of Solanaceae was identified and analyzed, and the response characteristics of the USP gene under mechanical damage and DON stress were predicted through the transcriptome. Screening multiple stress response genes laid a theoretical foundation for improving the quality and resilience of potatoes and provided a basis for potato genetic breeding.

## 2. Results

### 2.1. Genome-Wide Identification and Molecular Characterization of USP in Atlantic Potatoes

In this study, 108 StUSP genes were identified in Atlantic potatoes and renamed from *StUSP1* to *StUSP108*. The information about these StUSP genes and their corresponding proteins is shown in Table S1, including any renamed genes, their chromosomal physical locations, their protein lengths (aa), their molecular weights (MWs), their theoretical isoelectric points (pIs), their instability indexes, their aliphatic indexes, their total mean hydrophilic indexes, and their subcellular locations. The average length of StUSPs is 323 amino acids, and the protein length varies greatly from 134 aa (*StUSP18*) to 1433 aa (*StUSP101*). The average molecular weight was 35.86 kDa, and the molecular weight range was 14,610.77 Da (*StUSP18*)~160,203.58 Da (*StUSP101*). The theoretical isoelectric point (pI) ranges from 4.72 (*StUSP79*) to 10.06 (StUSP26, StUSP36), and the aliphatic index ranges from 71.23 (*StUSP56*, *StUSP63*, *StUSP73*) to 111.79 (*StUSP41*). The hydrophilic values ranged from −0.633 (*StUSP33*) to 0.211 (*StUSP18*), and the hydrophilic values varied greatly. The instability index of StUSPs ranges from 7.31 to 61.16 (>40 indicates protein instability; <40 indicates protein stability), including 51 stable proteins and 57 unstable proteins. Subcellular prediction showed that StUSPs were located in the cytoplasm (57), chloroplasts (27), nuclei (17), plasma membranes (3), peroxisomes (2), and cytoskeleton (2). These results suggest that universal stress proteins may adapt to different functional requirements by changing the length and physicochemical properties of amino acids.

### 2.2. Chromosome Localization and Gene Duplication Analysis of StUSPs in Atlantic Potato

**Figure 1:**
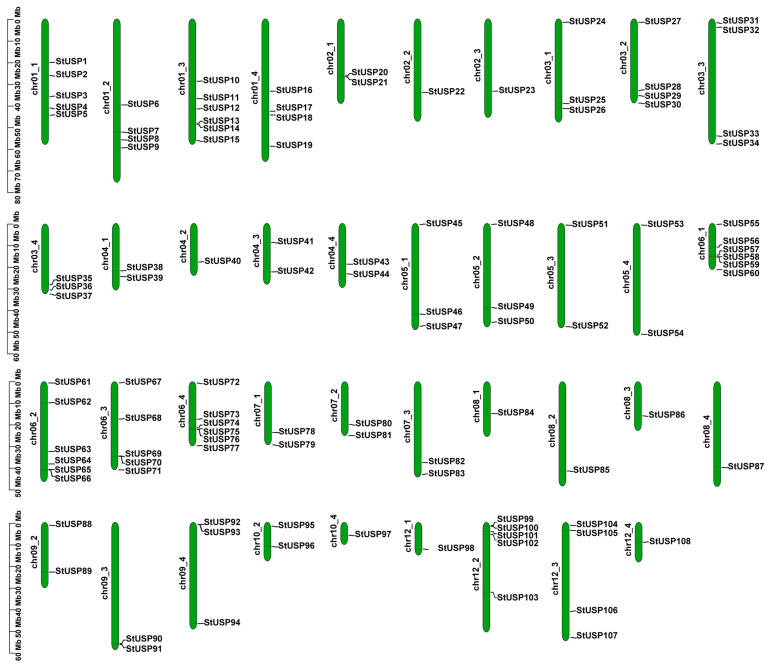
StUSP chromosome location map. The chromosome location map is drawn according to the gene location information on the chromosome. The location of genes can be estimated by referring to the scale on the left.

**Figure 2:**
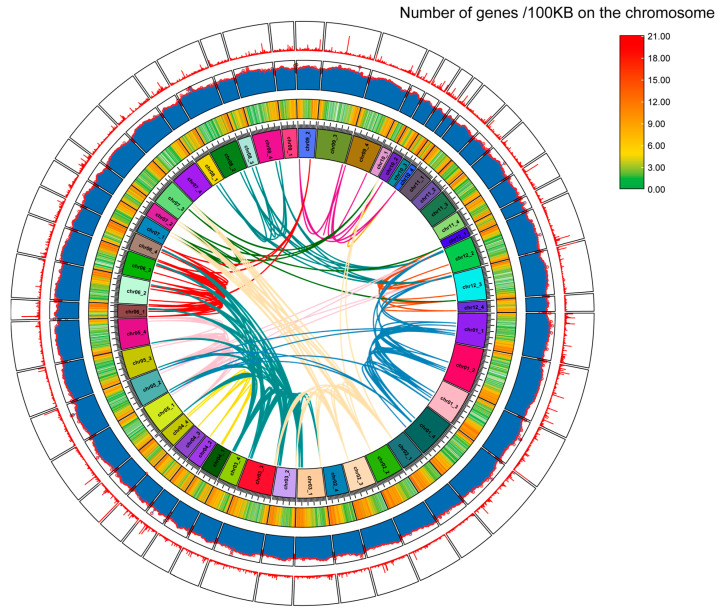
The synteny analysis of StUSPs in the potato genome. The rings from the inside out represent the chromosome skeleton, the gene density heat map (red for high gene density; green for low gene density), GC ratio histogram (the higher the GC ratio, the higher the histogram), and the N-ratio histogram (the more unknown bases the chromosome contains at this site, the higher the column). The collinear pairs of StUSPs genes in the potato genome are highlighted by colored lines.

Atlantic potato is an autotetraploid (2n = 4x = 48). A total of 108 StUSP genes were unevenly distributed on 39 chromosomes of potatoes (Figure 1). Chromosomes chr01_3, chr06_1, chr06_2, and chr06_4 contain the most StUSPs (six genes), and chromosomes chr01_1, chr06_3, and chr12_2 contain five StUSPs. In contrast, chromosomes chr02_2, chr02_3, chr04_2, chr08_1, chr08_3, chr08_4, chr10_4, chr12_1, and chr12_4 contain only one StUSP gene. However, the chromosomes of chr02_4, chr07_4, chr09_1, chr10_1, chr10_3, chr11_1, chr11_2, chr11_3, and chr11_4 do not contain the USP gene. Segmental duplication and tandem duplication are the key factors in gene family expansion. In our study, 103 StUSPs were found to be in the collinear region, with 202 pairs of segmental duplication. In addition, four tandem duplication gene pairs (*StUSP13/14*, *StUSP20/21*, *StUSP31/32*, and *StUSP99/100*) were identified on chromosomes chr01_3, chr02_1, chr03_3, and chr12_2 (Figure 2, Table S2). These results indicated that the StUSP gene family was mainly amplified through segmental duplication during the evolution of the Atlantic potato. The nonsynonymous substitution rate (Ka) and synonymous substitution rate (Ks) are the basis for evaluating whether collinear genes are subjected to selection pressure. A Ka/Ks value of 1 indicates natural selection; a Ka/Ks < 1 indicates purification selection; and a Ka/Ks > 1 indicates positive selection. The results showed that Ka/Ks ranges from 0.0745 to 1.4773, and most StUSP genes underwent a strong purification selection (Ka/Ks ratio < 1) after gene duplication events, suggesting that these genes have functional redundancy under certain conditions. In addition, three pairs of segmental duplication (*StUSP59/StUSP66*, *StUSP59/StUSP70*, and *StUSP61/StUSP72*) were affected by positive selection (Ka/Ks ratio > 1), suggesting that these genes changed proteins during the evolutionary process of stress selection (Table S3).

### 2.3. Phylogenetic Analysis of StUSPs

**Figure 3:**
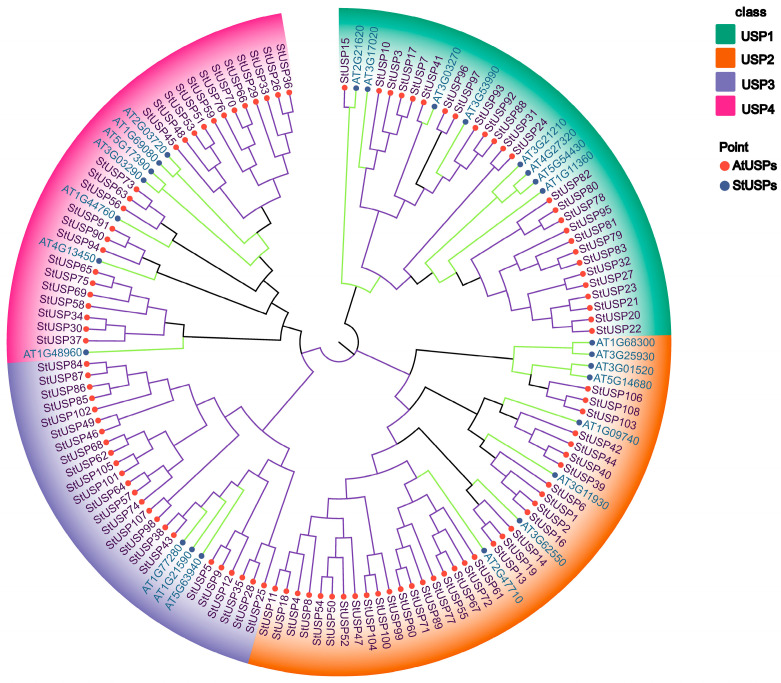
USPs’ protein phylogenetic analysis. Muscle was used to compare 26 AtUSPs and 108 StUPS sequences, and MEGA 7.0 was applied to construct the phylogenetic evolutionary tree using the neighbor-joining method. Shades of different colors indicate USP subgroup groupings. Different colored circles represent different species of USPs, with red circles representing potatoes and blue circles representing *Arabidopsis thaliana*.

In order to better understand the evolutionary relationship of StUSP family members, we selected 26 AtUSP protein sequences from the *Arabidopsis* genome database. Based on multiple comparisons of 108 common stress protein amino acid sequences, MEGA 7.0 was used to construct an adjacency phylogenetic tree by Neighbor-Joining(NJ) method, and the Poisson model was used to calculate the evolutionary distance (Figure 3). Phylogenetic analysis shows that the members of the StUSP can be divided into four subgroups. Among them, the USP2 subgroup contains 33 members, which is the largest subgroup. The USP4 subgroup contains a minimum of 24 members. The USP1 subgroup and the USP3 subgroup contain 26 and 25 StUSPs, respectively. The results showed that there is a common ancestor of the USP gene between potato and *Arabidopsis*, and specific copying and differentiation also occurred in the evolutionary process after separation.

### 2.4. Analysis of StUSPs’ Gene Structure, Domain, Conserved Motifs, and Secondary Structure

**Figure 4:**
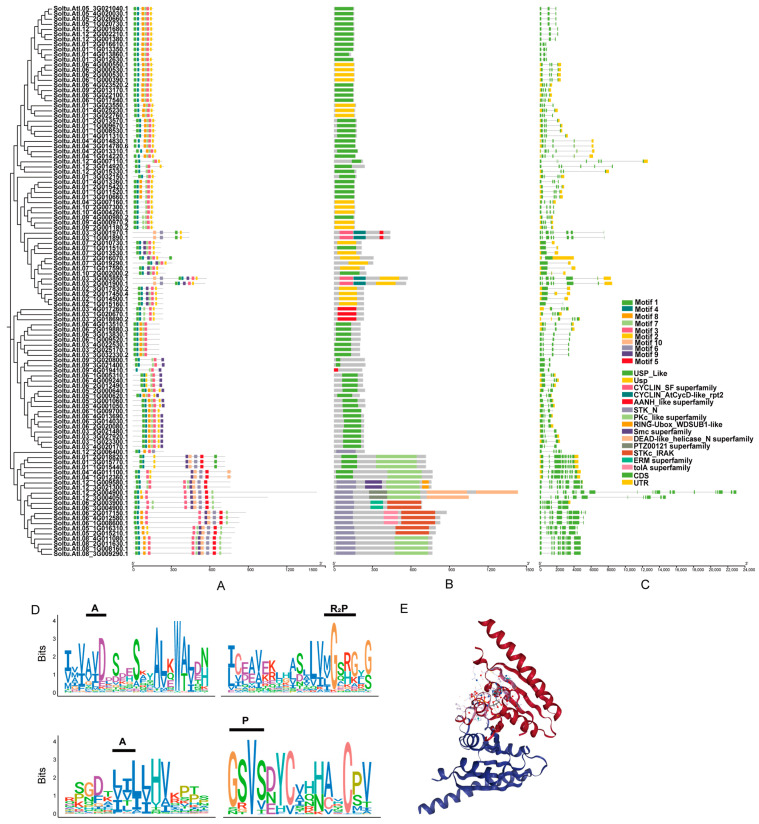
StUSP gene structure, protein motif, conserved domain analysis, and amino acid residue analysis of the StUSP domain: (**A**) The phylogenetic evolutionary tree of StUSPs is shown in the form of a tree map. The StUSPs’ motif is set to a maximum length of 25 and a minimum length of 6, with different colored rectangles representing conserved motifs 1–10 contained in StUSPs. (**B**) StUSPs’ conserved domain. Different colored rectangles represent different types of domains, and gray areas do not contain domains. (**C**) StUSPs’ gene structure. The gray lines represent introns, the yellow rectangles represent the 3′ or 5′ end non-coding regions, and the green rectangles represent exons. (**D**) MJ-0577 crystal structure model; data from Uniport.org. (**E**) The amino acid sequence of the USP domain in the StUSP logo diagram. The height of the amino acid represents the degree of conserved amino acid at this site. The higher the amino acid, the more it occurs at this site. The black notes above the sequence indicate ATP binding sites in the crystal structure of MJ-0577. ATP adenine (A), ATP phosphate (P), and ATP ribose (R).

An online MEME program was used to predict 108 StUSPs with a total of 10 conserved motifs (Figure 4). The length and conserved sequence of each motif are listed in Table S4. The composition and distribution of the motif are relatively conservative among members of the same subgroup (Figure 4A). Motif 1 and motif 4 are located at the N-terminal of most StUSP protein sequences, and motif 2 and motif 3 are located at the C-terminal. There are conserved glycine residues associated with the universal stress protein domain in these four motifs. Almost all USP1 and USP2 subgroups contain the following motifs: motif 1, motif 2, motif 3, and motif 4. These results show that the USP1 and USP2 subgroups have a relatively recent evolutionary history and a close phylogenetic relationship. The type and number of motifs in the same subgroup are similar, suggesting that the moiety pattern may be related to the function of the StUSP. Different subgroups usually have specific motifs, most of which are located at the C-terminal. For example, motif 5 is unique to the USP3 subgroup; motif 6 and motif 10 mainly appear in the USP4 subgroup; motif 8 mainly appears in the USP1 subgroup; and motif 9 mainly appears in the USP3 and USP4 subgroups.

To further explore the conserved domain of the StUSPs, the NCBI-Conserved Domain Database (https://structure.ncbi.nlm.nih.gov/Structure/cdd/wrpsb.cgi, accessed on 3 September 2023) was used to predict the StUSP (Figure 4B). Multiple sequence alignment of 108 StUSP genes showed that StUSP members had typical features of USP-conserved domains. The intron–exon distribution of StUSPs was analyzed by comparing the CDS sequence and the complete gene sequence (Figure 4C). A genetic structure analysis showed that the number of introns in StUSPs varied from 1 to 19. The largest number of introns was *StUSP101*, with 19 introns. It is worth noting that members of the same subgroup have similar structures, and most members have the same number of exons. A large number of StUSP genes (34 members, 31.48%) have a conserved gene structure of three introns and four exons, and the USP family members (26 members, 24.07%), with a conserved gene structure of two introns and three exons, account for the second largest proportion. The exon–intron structure pattern is very conservative among members of the same subgroup. The sequence similarity and the high similarity of the intron–exon structure indicate that the potato’s universal stress protein gene may have undergone gene replication events during evolution. The more special *StUSP105* has 14 exons and 13 introns, and *StUSP64* has 11 exons and 10 introns. The differences in exon–intron structure patterns between members of different subgroups of StUSPs may be caused by the deletion or acquisition of exons in the process of long-term evolution.

Ten conserved domains were found in the process of multiple sequence alignment (Figure S1). Through a comparison with MJ-0577 crystal (Figure 4D), four conserved domains related to ATP binding were found (Figure 4E). For example, in ATP adenine (A), ATP phosphate (P), and ATP ribosyl (R), in these domains, not all StUSPs contain glycine (G), and StUSPs are divided into two subgroups based on the presence or absence of a complete, ATP-binding, conserved-domain-specific binding sequence (G-2X-G-9X-G [S/T]). One domain contained ATP binding sites (55) and the other domain did not contain ATP binding sites (53). In the StUSPs’ secondary structure, on average, 117 amino acids (aa) constitute the α helix, accounting for 36.37% of the total secondary structure. On average, 53 amino acids (18.38%) form the extended chain, and 18 amino acids (5.36%) form the beta fold. A total of 133 amino acids constitutes a random crimp, accounting for 40.11% (Table S5). The results show that random crimping and the α-helix play an important role in the formation of the tertiary structure of StUSPs.

### 2.5. Analysis of Cis-Acting Elements of StUSPs

**Figure 5:**
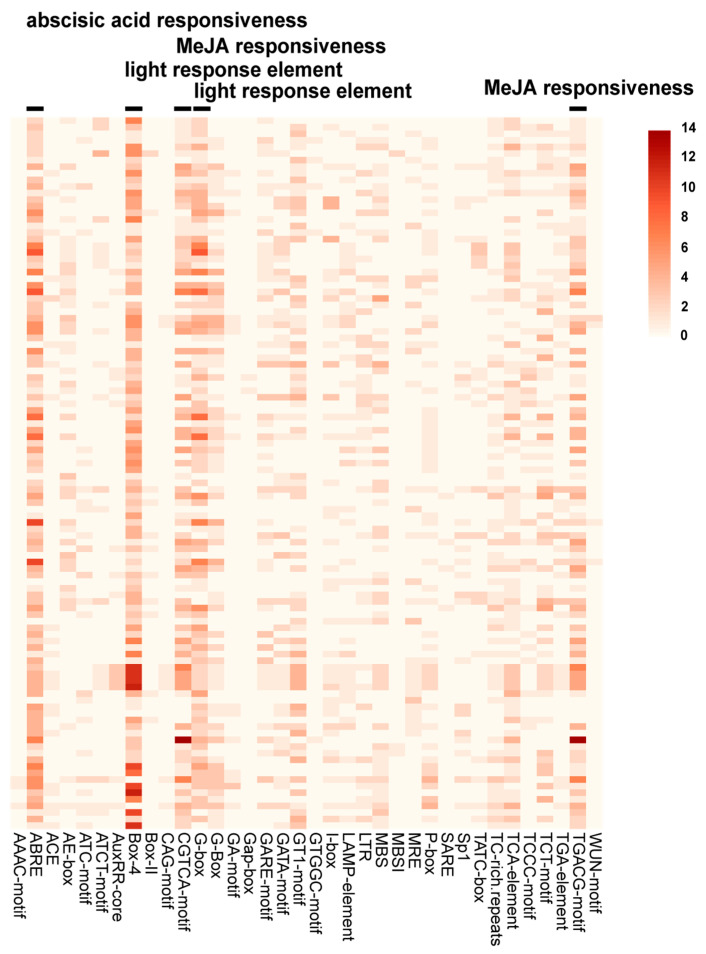
Predicted cis-acting elements in the promoter regions of Atlantic potato StUSP genes. The color code on the right shows the number of cis-acting elements and notes the response types of the more abundant elements.

Promoter cis-acting elements play a key role in gene expression initiation. We analyzed the cis-acting elements of the 108 StUSP gene promoter regions (Table S6). The results showed that the cis-acting elements of the StUSP gene promoter region involved in the stress response were mainly divided into hormone response elements, light response elements, environmental-stress-related elements, and site-binding elements. The number of methyl jasmonate response elements are 482, making this the largest response element. The second most numerous is the abscisic acid response element, with 364 elements. In addition, there are auxin response elements, gibberellin response elements, and salicylic acid response elements. All members of the StUSP family have more abscisic acid response elements and methyl jasmonate response elements in the promoter region, among which ABRE response elements are the most common in the StUSP gene promoter (Figure 5). The results indicated that the expression of the StUSP gene may be regulated by plant hormones and abiotic stress during potato growth, which improves the plant’s tolerance to stress.

### 2.6. StUSP Gene Collinear Analysis

**Figure 6:**
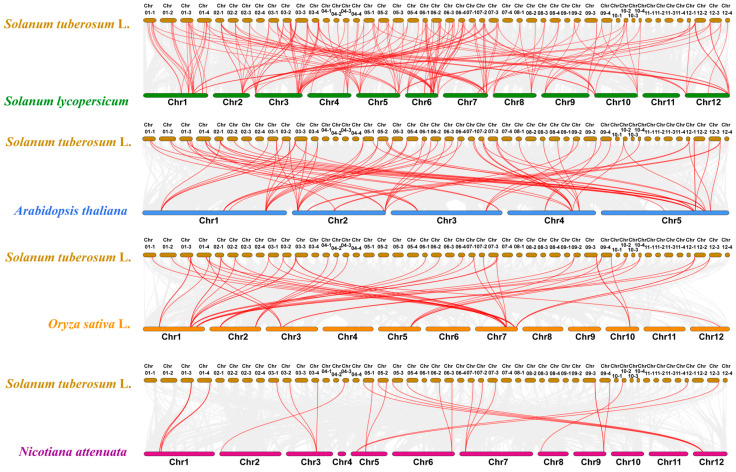
Homology analysis of USP genes between the Atlantic potato and four other plants. The gray lines represent gene blocks in the Atlantic potato that are homologous to other genomes. The collinearity relationship between potato and *Solanum lycopersicum*, *Arabidopsis thaliana*, *Oryza sativa* L., and *Nicotiana attenuata* USP genes was analyzed with MCscanX, and collinear gene pairs are highlighted with red lines. The gray lines represent gene blocks in the Atlantic potato that are homologous to other genomes.

Comparative genomics is a powerful tool for quickly identifying and locating unknown genes through comparing genes and genome structures. According to the previous gene count, chromosome localization, and phylogenetic tree analysis, the StUSPs gene was relatively conserved in the evolution of potatoes. In order to reveal the similarity of StUSPs with homologous genes of other species, and further study the evolutionary relationship of potato StUSPs, we took the potato StUSPs sequence as the core and constructed a collinear relationship of StUSPs with four representative plants (*Solanum lycopersicum*, *Arabidopsis thaliana*, *Oryza sativa* L., and *Nicotiana attenuate*). The results showed that the logarithm of the collinear genes of potato and tomato was 184 pairs, the logarithm of the collinear genes of potato and *Arabidopsis* had 78 pairs, the logarithm of the collinear genes of potato and rice was 53 pairs, and the logarithm of the collinear genes of potato and coyote tobacco was 26 (Figure 6, Table S7). The collinearity between potato and tomato is significantly higher than that of the other three plants. The number of collinear gene pairs among Solanaceae plants varies greatly. The collinear gene pairs of potatoes and tomatoes are mainly concentrated on the chr1, chr3, chr5, chr6, and chr7 chromosomes. The collinear gene pairs of potato and coyote tobacco are mainly concentrated on the chr1 and chr12 chromosomes; the collinear gene pairs of potato and monocotyledonous model plant rice are mainly concentrated on the chr1 and chr7 chromosomes; and the collinear gene pairs of the model plant *Arabidopsis* are mainly concentrated on chr1, chr4, and chr5. The results show that the StUSPs genes on the potato chr04_2 and chr08_2 chromosomes may mainly come from tomatoes. These results show that the Atlantic potato StUSP gene family has expanded and evolved through genome-wide replication.

### 2.7. Analysis of Expression of StUSPs in Response to Mechanical Damage and DON Stress

**Figure 7:**
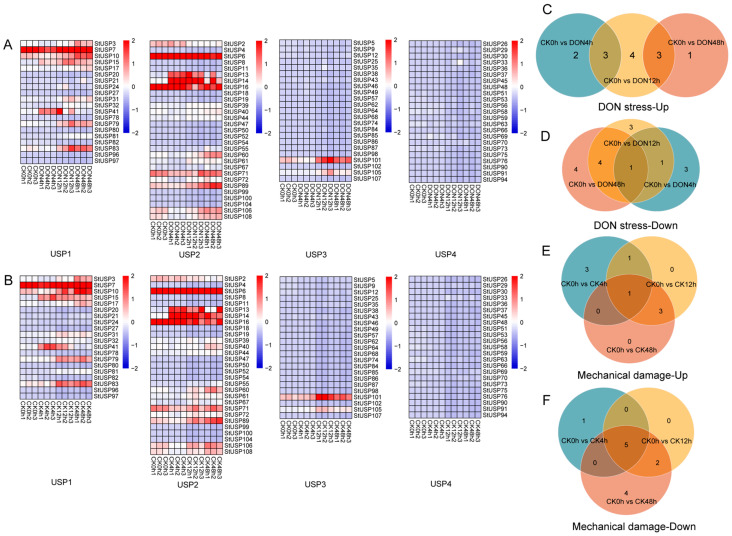
Differential expression of the StUSP gene under DON stress and mechanical damage. Heat map of StUSP gene expression in potatoes treated with DON stress (**A**) and mechanical damage (**B**). The heat map is based on the StUSP subgroup classification. (**C**–**F**) Venn diagram of the number of DEGs in USP1 and USP2 subfamilies compared with control groups under DON stress and mechanical damage, with (**C**,**E**) indicating up and (**D**,**F**) indicating down.

RNA-Seq was used to study the gene expression patterns of StUSPs after mechanical damage and DON stress at different stress times (0 h, 4 h, 12 h, 48 h). The StUSP fragments per kilobase of exon model per million mapped fragments (FPKM) values of *StUSPs* for each treatment are shown in Table S8, excluding genes with no expression or low expression levels, and finally the expression patterns of 96 StUSP genes are obtained (Figure 7A,B). The expression levels of StUSPs in the four subgroups are different under different stresses. Among them, the StUSPs of the USP3 and USP4 subgroups mainly show a low level of expression. However, mechanical damage and DON stress have a significant inducing effect on the StUSPs of the USP1 and USP2 subgroups, and the gene induction effect is closely related to the induction time. These results show that the StUSPs gene plays an important role in responding to mechanical damage and DON stress. Among the 108 members of the potato StUSP gene family, more than half of the genes are highly expressed during at least one stage of potato adversity stress. About one half of the gene expression level is basically unchanged.

In order to screen for the StUSP genes involved in multiple stress responses, FPKM values were used to estimate the expression level of StUSP genes. The differential expression genes (DEGs) caused by DON stress and damage stress are shown in Venn diagrams. After 4 h of DON treatment, five genes in StUSPs were upregulated and five genes in StUSPs were downregulated. After 12 h of DON treatment, 10 genes in StUSPs were upregulated and 9 genes in StUSPs were downregulated (Figure 7C,D). Through the analysis of the pre-stress response results, after 4 h of mechanical damage treatment, five genes in StUSPs were upregulated and six genes in StUSPs were downregulated. After 12 h of mechanical damage treatment, five genes in StUSPs were upregulated and seven genes in StUSPs were downregulated (Figure 7E,F). In order to determine the multiple stress response genes, we screened the upregulated genes involved in the early adversity response. During this process, we screened six StUSP candidate genes in response to mechanical damage and five StUSP candidate genes in response to DON stress.

### 2.8. StUSP K-Means Analysis

**Figure 8:**
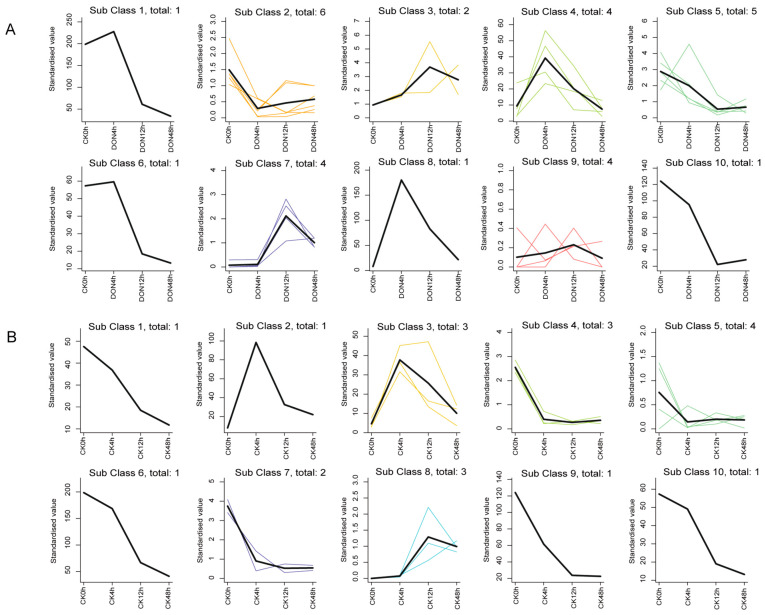
K-means clustering analysis of DEG profiles. The black line represents the expression model. The other colorful lines are the expression profiles of each DEG. The x-axis represents different treatment times. The y-axis represents the standardized value. DON stress (**A**) and mechanical damage (**B**).

The DEGs in each treatment group were analyzed with K-means clustering, and the differential genes were grouped into 10 clusters for each stress treatment (Table S9). Among them, the 29 differential genes caused by DON stress showed a trend of high expression for 4 h in classes 1, 4, 6, and 8, while classes 3 and 7 showed a trend of high expression for 12 h (Figure 8A), and the 20 differential genes caused by mechanical damage showed a trend of high expression for 4 h in classes 2 and 3, and class 8 showed a trend of high expression for 12 h (Figure 8B). In order to screen the StUSP genes involved in the response to multiple stresses, the clustering groups obtained from the two groups were intersected. At the same time, the gene range was further narrowed with reference to Venn analysis, and finally, three StUSP genes (*StUSP13*, *StUSP14*, and *StUSP41*) involved in the response to mechanical damage and three StUSP genes (*StUSP13*, *StUSP14*, and *StUSP15*) involved in the response to DON stress were selected.

### 2.9. Analysis of Gene Coexpression

**Figure 9:**
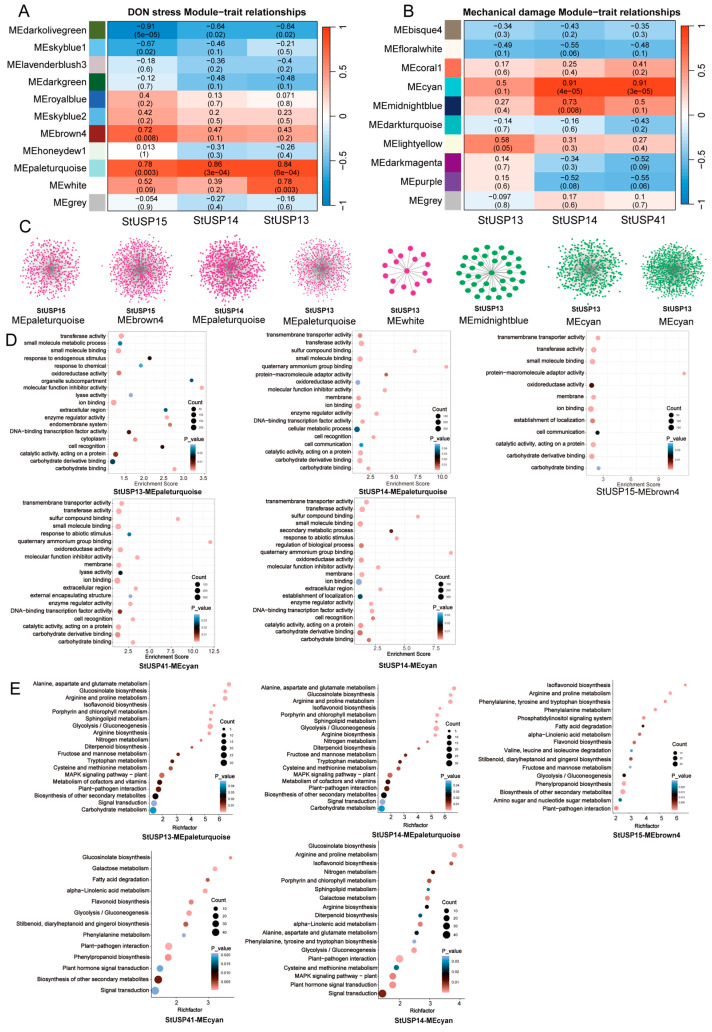
WGCNA of differential gene coexpression networks. DON stress (**A**), and mechanical damage (**B**). Correlation between modules’ central genes and corresponding *p*-values (in parentheses). The colored panel on the left shows the individual module information. The color code on the right shows the correlation between modules’ central genes. (**C**) Eight modules with a correlation of greater than 0.7 to the central gene construct a gene coexpression network, where dots represent genes and lines indicate coexpression relationships. (**D**) Bubble map of the Go enrichment analysis of coexpressed gene sets of key modules, showing the path with *p*-value < 0.05. (**E**) Bubble map of KEGG enrichment analysis of coexpressed gene sets of key modules, showing the path with *p*-value < 0.05.

Coexpression analysis can help find genes with similar expression patterns, which is of great significance in the study of plant stress response mechanisms. These genes may be functionally closely related or participate in the same signaling pathway or physiological process. In order to elucidate the role of the StUSP gene in the potatoes’ response to multiple stresses, in this study, we used the Weighted Correlation Network Analysis (WGCNA) method, combined with the RNA-Seq data of seven samples, which uses a dynamic shear algorithm to cluster genes and divide them into modules. By calculating the feature vectors of each module and combining similar modules, a coexpression network was constructed centered on the above three differentially expressed DON stress response genes (*StUSP13*, *StUSP14*, and *StUSP15*) and three mechanical damage response genes (*StUSP13*, *StUSP14*, and *StUSP41*) (Figure 9A). As shown in the figure, we obtained a total of eight coexpression modules and used the central gene and its interacting differential genes to map the gene interaction network (Figure 9B). Among them, in the DON stress response, the MEBrown module centered on *StUSP15* has the largest network (742 genes), followed by the MEpaleturquoise module centered on *StUSP14*, with 731 coexpressed genes. The MEwhite module network centered on *StUSP13* is the smallest (16 genes). Among the mechanical damage response modules, the MEcyan module network centered on *StUSP14* is the largest (863 genes), followed by the MEcyan module centered on *StUSP41*, with 468 coexpressed genes, and the MEmidnightblue module network centered on *StUSP14* is the smallest (36 genes). This coexpression network further proves the complex function and potential role of the StUSP gene family in multiple stress responses.

In order to explore the biological processes that these coexpressed genes may be involved in, we selected five modules with a large number of coexpressed genes (*StUSP13*, *StUSP14*, *StUSP15*, and *StUSP41*) that are involved in a variety of stress responses for gene-set enrichment analysis (Figure 9C). There are a total of 11 significantly enriched GO terms. Common GO terms include transferase activity, molecular function inhibitor activity, enzyme regulator activity, small-molecule binding, catalytic activity, carbohydrate-derivative binding, ion binding, carbohydrate binding, DNA-binding transcription factor activity, oxidoreductase activity, and cell recognition. This shows that these four genes may play an important role in binding activity and catalytic activity in response to multiple stresses (Figure 9D). In response to DON stress, the coexpressed gene networks of *StUSP13* and *StUSP15* are mainly enriched in GO terms to regulate responses to an endogenic stimulus, small-molecule metabolic processes, and responses to chemicals. The *StUSP14* coexpressed gene network is significantly enriched in GO terms to regulate transmembrane transporter activity, cellular metabolic processes, and other biological processes. We suspect that these genes may respond to DON stress by regulating the ion balance and cell metabolism. Among the genes that respond to injury stress, the *StUSP41* coexpressed gene network is significantly enriched in response to abiotic stimulus and transmembrane transporter activity. The *StUSP14* coexpressed gene network is significantly enriched in biological processes such as transmembrane transporter activity, response to abiotic stimulus, regulation of biological process, and secondary metabolic process. This suggests that our genes may respond to the damage caused by mechanical damage to plants by regulating transmembrane transporter activity and secondary metabolic pathways. Interestingly, the *StUSP13*, *StUSP14*, *StUSP15*, and *StUSP41* genes have the same expression patterns in the two stresses, so we speculate that these genes are closely related to stress response.

Kyoto Encyclopedia of Genes and Genomes (KEGG) enrichment analysis (Figure 9E) shows that, during the DON stress response, the genes coexpressed with *StUSP13* are significantly enriched in the biosynthesis of isoflavones, the plant–pathogen response, plant hormone signal transduction, the mitogen-activated protein kinase (MAPK) signaling pathway, and other metabolic processes; the genes coexpressed with *StUSP14* are significantly enriched in the amino acid metabolism process, the plant–pathogen response process, the starch and sucrose metabolism process, the MAPK signaling pathway, and secondary metabolite synthesis. The biosynthesis of flavonoids, the plant–pathogen response, phenylpropane biosynthesis, the amino acid metabolism, and the secondary metabolite synthesis processes of genes coexpressed with *StUSP15* are significantly enriched. During the mechanical damage response, the genes coexpressed with *StUSP14* are significantly enriched in the biosynthesis of flavonoids, the biosynthesis of phenylpropane, the plant–pathogen response, plant hormone signal transduction, and the synthesis of secondary metabolites, and the genes coexpressed with *StUSP41* are significantly enriched in the biosynthesis of flavonoids, the biosynthesis of phenylpropane, the plant–pathogen response, the biosynthesis of isoflavones, and the synthesis of secondary metabolites.

### 2.10. qRT-PCR Verifies StUSP Gene Expression

**Figure 10:**
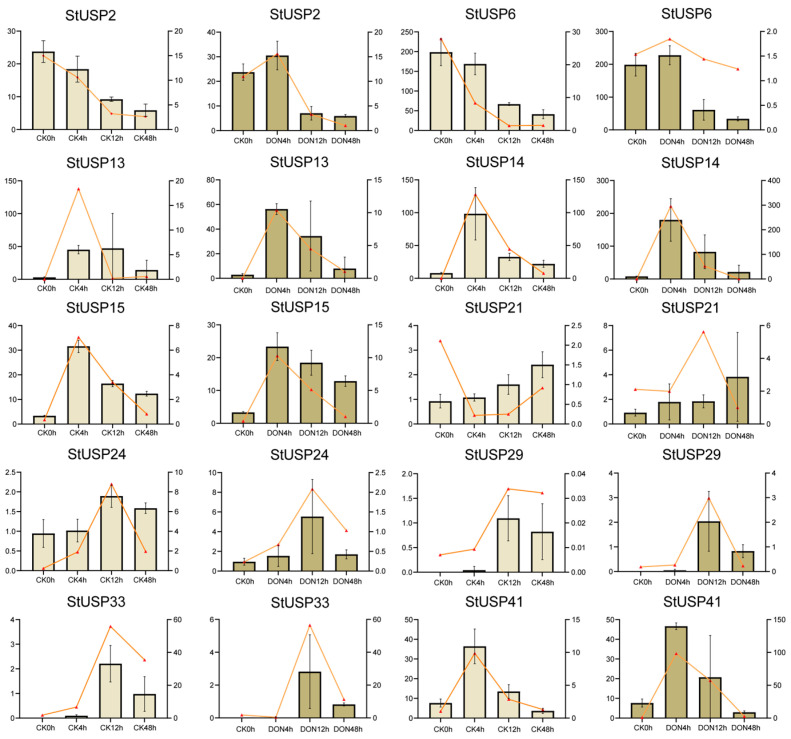
Gene expression levels are based on FPKM and qRT-PCR. CK0h represents the control group; CK4h, CK12h, and CK48h represent 4 h, 12 h, and 48 h after mechanical damage treatment, respectively. DON4h, DON12h, and DON48h stand for 4 h, 12 h, and 48 h after treatment with DON, respectively. The bar chart and the left y axis represent FPKM, and the line chart and the right y axis represent q-RT-PCR.

Based on RNA-seq, we speculated that 10 StUSP genes (*StUSP2*, *6*, *13*, *14*, *15*, *21*, *24*, *29*, *33*, and *41*) in potatoes are involved in the potatoes’ adversity response process. We used quantitative real-time PCR (qRT-PCR) to quantitatively determine the expression of *StUSPs* at different mechanical damage and DON stress levels. β-actin protein was used as an internal reference gene for gene normalization. The expression trends of genes in RNA-Seq and qRT-PCR are basically similar. Some of the differences in gene expression trends may be due to experimental errors in RNA-Seq or qRT-PCR. During multiple stress responses, the expression of genes *StUSP13*, *StUSP14*, *StUSP15*, and *StUSP41* is relatively high at 4 h. The expression of genes *StUSP24*, *StUSP29*, and *StUSP33* is relatively high at 12 h. The gene expression levels of *StUSP13*, *StUSP14*, and *StUSP41* are significantly induced in the response to multiple stresses. In response to multiple stresses, the expression of the *StUSP14* gene is the highest among all genes. This gene exhibits significant characteristics in response to mechanical damage and DON stress (Figure 10). The primers are listed in the Supplementary Materials (Table S11).

## 3. Discussion

USPs are widely distributed in various organisms and are an important part of the defense system. Through cellular tolerance mechanisms, organisms are endowed with tolerance to multiple stress responses [22]. In plants, USPs are directly involved in many physiological and metabolic activities and play an important role in regulating plant growth and development [40], resisting environmental interference [41], and responding to biological and abiotic stresses [42]. A large number of USPs have been identified in plants that respond to abiotic stress, such as *Arabidopsis* [29], cotton [43], barley (*Hordeum vulgare*) [19], rice [24], wheat (*Triticum aestivum*) [27], and Pigeonpea (*Cajanus cajan*) [44], but few USPs have been identified with functional characteristics. The identification of the total StUSP genes and the response pattern of the StUSP genes under adverse conditions are important steps in understanding their downstream signaling and determining their regulatory pathway. These proteins have not been reported to have specific functions in potatoes, so the lack of a genome-wide analysis of genes containing USP domains hinders a comprehensive understanding of the evolutionary history and biological functions of potato StUSPs. Among the large number of StUSP gene members, we prioritized the StUSP gene that responds to adversity. A genome-wide analysis of the USP gene family was carried out in many species, and a single plant genome usually contains 20–50 members [45], including 44 members in *Arabidopsis* [29], 85 in wheat [27], 42 in tomato [46], 21 in grape (*Vitis vinifera*) [23], 32 in Dan-Shen Root (*Salvia miltiorrhiza*) [47], etc.

A total of 108 potato StUSP genes were identified in this study, more than those in *Arabidopsis*, rice, tomato, and coyote tobacco. As a homologous tetraploid, the genome size of the Atlantic potato is 2.57 Gb [48], while the genome sizes of *Arabidopsis* [49], rice [50], tomato [51], and coyote tobacco [52] are 116 Mb, 363 Mb, 802 Mb, and 2.254 Gb, respectively. In this case, there may be a direct correlation between the number of USP genes and genome size in these plants. 

In our study, based on phylogenetic analysis and collinear analysis, it was found that the members of the AtUSP gene family of *Arabidopsis thaliana* can divide the USPs in potatoes into four subgroups. Most subgroups contain AtUSP members from *Arabidopsis*. However, in the USP3 subgroup, some StUSPs are not divided into clusters with AtUSPs, which indicates that they may not share a common ancestor with *Arabidopsis*. In addition, the USP gene family can also undergo species-specific differentiation after separation. The evolutionary relationship between species is revealed by the number of collinear gene pairs. In order to reveal the similarity and evolutionary relationship between USPs of different species, the collinear relationship between four other representative plants and StUSP homologous genes was analyzed. The results showed that StUSPs had the highest homology with USPs in tomatoes and a relatively low homology with USPs in coyote tobacco. Studies have shown that the differentiation of homologous genes caused by gene duplication can promote the production of new characteristics or new gene functions. Over time, the functions of gene family members also changed. It is reported that polyploidy and gene-region-specific duplication (tandem duplication and segmental duplication) are important mechanisms for the generation and expansion of plant gene families [53]. A total of 4 pairs of tandem repeat genes and 202 pairs of segmental duplication genes were identified in the potato StUSP gene family, which indicates that segmental duplication events are the main source of amplification of the StUSP gene family in potatoes. This result may be due to the homologous tetraploid of Atlantic potatoes.

The evolution of gene families depends to a large extent on the arrangement of the gene structure. The different nucleotide sequence lengths of StUSPs between different subgroups, and even different members of the same subgroups, indicate the complexity of the Atlantic potato genome. The molecular weight and isoelectric point values of StUSPs, and other physical and chemical properties, are also different between family members, which indicates that there are differences in their functions. In addition, the StUSPs contain 10 conserved motifs of different compositions, and members of the same subgroup contain similar motif types and similar numbers of motifs, proving the conserved nature and diversity of the potato StUSP gene family. The composition of StUSPs’ exons and introns varies greatly between the subgroups, but the genetic structure of members of the same subgroups is very different, which shows that the StUSP genes in the subgroups are highly conserved. The gene structure of the members of the USP1 and USP2 subgroups is highly similar. Most gene members have four exons and three introns, as well as three exons and two introns splicing patterns. Such genes account for more than 50% of the entire StUSP gene family. The members of the USP4 subgroup have the largest number of introns and still exhibit low expression patterns under multiple stresses. We speculate that the genetic structure differences between the members are large because intron insertion events occur only very occasionally in the domain region of StUSPs, and this intron pattern remains conserved during long-term evolution. The number of introns is positively correlated with the time required for gene transcription and translation. The smaller the number of introns, the faster the gene is expressed when the environment changes; therefore, they perform a more effective function [54]. The results showed that *StUSP13*, *StUSP14*, *StUSP15*, and *StUSP41* all contain splicing patterns of four exons and three introns. Therefore, we speculate that these two genes may have a more important function in responding to multiple stresses. The StUSP is composed of a highly conserved USP family protein domain, and closely evolutionarily related proteins are more similar in the composition of the conserved domain. In addition to the characteristic domains, most proteins also contain STK_N, STKc_IRAK, CYCLIN_AtCycD-like_rpt2, and PKc_like superfamily domains. The structural diversity of protein domains may be one of the reasons that StUSPs have multiple physiological and metabolic functions.

The cis-regulatory elements of the promoter play a key role in initiating gene expression, and the presence of different cis-regulatory elements in the promoter sequence may lead to different gene expression patterns [55]. Through the analysis of cis-acting elements, genes with specific functions related to plant adversity responses can be effectively screened in plants. In this study, a total of 40 cis-acting element sequences related to stress response were identified in the promoter region of the StUSP gene, and each member contains at least 10 or more elements, which indicates that the expression of the StUSP gene family is affected by environmental stress. Among these elements, 10 are related to plant hormone response, 24 are related to light response elements, and 3 are related to stress-regulatory elements. The diverse cis-regulatory elements in the promoter region of the StUSP gene may also reflect functional differentiation at the transcriptional level.

Previous reports have shown that the AtUSP promoter can be strongly induced by a variety of abiotic stress sources and can produce multiple stress tolerances [31]. In another study of cotton plants, it was found that the promoter of the USP gene responds to ABA, salt stress, and heavy metals [56]. In addition, the wild tomato USP gene was induced by ABA, injury, and cold stress [39]. This shows that the regulatory elements of the StUSP gene play an important role in the potato’s defense against different environmental stresses and can effectively regulate the potato’s cell tolerance after environmental stress and maintain a normal growth state.

In a variety of plants, USP genes have been shown to be related to biological and abiotic stress. For example, the function of *SlRd2* in tomatoes regulates SlCipk6-mediated ROS production [57]. A universal stress protein MsUSPA is involved in regulating the synthesis of hormones and secondary metabolites. The overexpression of *MsUSPA* can increase the activity of antioxidant enzymes and reduce the accumulation of ROS [58]. In cottons, *GhUSP1* and *GhUSP2* are involved in the response to salt stress [59]. In this study, gene expression data were used to analyze the functional effects of the StUSP gene and identify the different expression patterns of the StUSP gene under different abiotic stresses. The results showed that there is a functional differentiation of the StUSP gene between different subgroups. The USP3 and USP4 subgroups are insensitive to stress response, and the response of the USP1 and USP2 subgroups to stress is closely related to the response time. Generally speaking, genes with similar structures are gathered in the same superfamily, and these genes may have similar biological functions. According to the expression pattern of *AT2g47710* (*AtUSP10*), it was significantly induced under salt stress and osmotic stress conditions [60]. In the process of identifying drought-responsive universal stress proteins in leafy plants, *AT3g62550* was considered to be involved in drought stress response [61]. Studies have shown that the accumulation of At2g21620 protein under abiotic stress is significantly increased [62], and *At3g03270* can be induced by ERF-VII protein RAP2.12 (related to Apetala 2.12) and regulate the production of ROS in *Arabidopsis* [63]. Therefore, homologous clusters in the USP1 and USP2 subgroups may have similar functions. In this study, StUSP13 and StUSP14 in the same cluster as *Arabidopsis* AT2g47710 and AT3g62550, StUSP15 in the same cluster as At2g21620, and StUSP41 in the same cluster as At3g03270 showed similar response patterns under mechanical damage and DON stress. These genes may be involved in the response to mechanical damage and toxic stress.

In this study, there are different expression patterns between StUSP genes, and it is inferred that the functional differentiation of family gene members should also coexist. For example, *StUSP13* and *StUSP14* belong to tandem repeat gene pairs. The results of the study showed that *StUSP13* and *StUSP14* were significantly upregulated in the early stages of the two stress treatments. The expression levels of these two genes were particularly high after 4 h of treatment, while *StUSP19* was repeated as a segmental of *StUSP13* and *StUSP14* gene pairs and showed low expression levels at all timepoints during the two stress treatments. In theory, there are nonfunctional, neofunctional, and subfunctional evolutionary directions for repetitive genes. It can be inferred that, during the long process of evolution, *StUSP19* degenerated and lost its original function.

The results of RNA-Seq analysis showed that, under mechanical damage and DON stress, the expression of *StUSP15* and *StUSP41* of the USP1 subgroup and *StUSP13* and *StUSP14* of the USP2 subgroup were significantly induced, and under 4 h of stress, the gene expression level of *StUSP14* was the highest. By analyzing the differential gene conditions and gene expression levels under stress, it was found that these genes showed significant responses during multiple stress responses and showed positive responses to adversity. It is reported that USPs are mainly involved in the regulatory process related to stress response [64,65,66]. According to the expression law of USPs in other organisms, StUSPs can be used as a key resistance gene for functional research. It is speculated that they may have the regulatory effect of enhancing the potato’s resistance to fungal infection and maintaining normal life activity. In order to further understand the functions of *StUSP13*, *StUSP14*, *StUSP15*, and *StUSP41*, we used these as the central genes in the WGCNA by screening the coexpression network module and then performing a gene enrichment annotation and KEGG enrichment analysis on the coexpressed genes in the module. The results of the gene set enrichment analysis show that the genes coexpressed with them in the response to multiple stresses are significantly enriched in terms of their regulating ion binding, transmembrane transporter activity, and cell response to stimulation. This shows that different regulatory factors and regulatory gene networks play an important role in specific biological functions. KEGG enrichment analysis showed that *StUSP13* and *StUSP14* are mainly involved in plant hormone signal transduction, plant response to pathogens, and MAPK signal transduction by regulating coexpressed genes, while *StUSP15* and *StUSP41* are mainly involved in phenylpropane biosynthesis, flavonoid biosynthesis, isoflavone biosynthesis, and the synthesis of secondary metabolites in response to the damage caused to potatoes in adverse conditions. The metabolism and signal transduction processes of these substances enhance their defense against adversity by adjusting the cell’s own tolerance to environmental stress in response to the production of reactive oxygen species (ROS), effective protein turnover, and changes in Ca^2+^ cell levels following mechanical damage.

The results of the study show that *StUSP13*, *StUSP14*, *StUSP15*, and *StUSP41* are regulatory genes of the potato StUSP gene family in response to multiple stresses. In addition, the expression patterns of 10 StUSPs genes under mechanical damage and DON stress were analyzed by qRT-PCR. In addition to *StUSP2*, *StUSP6*, and *StUSP21*, a total of seven genes are significantly regulated by the two stress treatments, suggesting that StUSPs may be involved in the crosstalk of different signaling pathways under stress. This study systematically analyzed the biological characteristics and expression patterns of USPs. These analyses not only help to screen valuable candidate StUSPs genes for further functional research but also have important implications for agricultural production and the genetic improvement in potato crop resistance.

## 4. Material and Methods

### 4.1. Identification of the USP Gene

In this study, the Hidden Markov Model (HMM) of the conserved-domain USP (PF00582) of the universal stress protein was downloaded from the PFam website (https://pfam.xfam.org/, accessed on 12 August 2023) [67], and HMMER3.0 was used (http://hmmer.org/download.html, accessed on 27 August 2023) to search for USP candidate gene family members [68]. We obtained the protein sequence of AtUSP from the *Arabidopsis* database TAIR (http://www.arabidopsis.org/, accessed on 12 August 2023) and compared the sequences of candidate members using the BLASTP method (E ≤ 1 × 10^−5^) [45]. After manually removing redundant proteins, NCBI-CDD [69] was used to retrieve candidate protein sequences containing complete USP domains in the genome of the Atlantic potato. The genes were renamed according to their position on the chromosome, and they were named *StUSP1*–*StUSP108*.

### 4.2. Physical Mapping of Genes on Chromosomes and Collinearity Analysis

The start and end locations of all genes on 48 chromosomes were obtained from the potato database (http://spuddb.uga.edu, accessed on 28 August 2023), and *StUSPs* collinearity analysis was performed using MCScanX in TBtools software (v1.106) [70]. The presence of segmental duplication and tandem duplication events was defined based on the physical location of genes in chromosomes [71], and the ratio of nonsynonymous substitution to the synonymous substitution (Ka/Ks) of duplicate gene pairs was determined by KaKs_Calculator (v2.0) [72]. Dual Synteny Plot in TBtools (v1.106) was used to analyze the collinearity of StUSPs with other species (*Arabidopsis*, rice, coyote tobacco, and tomato) [73]. TBtools (v1.106) was used to visualize the results [74].

### 4.3. Multiple Sequence Alignment and Phylogenetic Analysis 

In order to explore the evolutionary relationship of the USP gene family, the muscle program in MEGA 7 [75] software was used to perform multiple sequence comparisons of USP proteins between potato and *Arabidopsis thaliana*, and the phylogenetic tree was constructed by the neighbor-joining method. Maximum likelihood (ML) was used with 1000 bootstraps and other default parameters. ITOL (https://itol.embl.de/, accessed on 29 August 2023) was adopted to beautify the evolutionary tree.

### 4.4. Exon–Intron, Conserved Motif Analysis, and Cis-Acting Element Analysis

The StUSP gene structure (exon–intron) was defined by TBtools software, and the conserved motif of the StUSP protein was obtained from the online MEME program (https://meme-suite.org/, accessed on 28 August 2023) [76]. The following parameters were used: minimum width set to 6 bp, maximum width set to 25 bp, and maximum number of motifs set to 10. The structure and conserved domain of the StUSP gene were visualized by TBtools. The upstream 2000 bp sequence of the start codon of each StUSP gene was obtained from the potato genome through the Plant CARE database for StUSP cis-acting elements in a sequence of gene promoters for online retrieval [77].

### 4.5. Analysis of Physical and Chemical Properties, Secondary Structure, and Subcellular Localization

Amino acid number, molecular weight, isoelectric point, hydrophobic index, and other information about StUSP members was analyzed by ExPASy (https://www.expasy.org/, accessed on 28 August 2023) [78]. WoLF PSORT (https://wolfpsort.hgc.jp, accessed on 4 September 2023) was used to predict protein subcellular localization [79]. SOPMA (https://npsa-prabi.ibcp.fr/cgi-bin/npsa_automat.pl?page=npsa_sopma.html, accessed on 28 August 2023) was used to predict the secondary structure of the StUSP protein [80].

### 4.6. Plant Materials and Stress Treatment

To study the dynamic response of the StUSP gene family to multiple stresses, Atlantic potato tubers were sprayed with 5 ng/mL DON aqueous solution (purchased from Fermentek, Jerusalem, Israel) at 4 h, 12 h, and 48 h (recorded as DON4h, DON12h, and DON48h, respectively) to serve as DON stress groups. Potato tubers were scratched 1 cm deep with sterile blades at 4 h, 12 h, and 48 h (recorded as CK4h, CK12h, and CK48h, respectively) as mechanical damage groups, and samples were taken at 0 h under sterile water treatment (recorded as CK0h) as a control group. All samples of potato tubers were frozen in liquid nitrogen and stored at −80 °C. All groups of samples were bioreplicated three times. At the same time, the normal-growing potato plants were treated with DON stress and mechanical damage, and the sampling time was the same as above. All samples were frozen in liquid nitrogen and stored at −80 °C, and the samples were bioreplicated three times.

### 4.7. Analysis of RNA-Seq and StUSP Gene Expression

The potato transcriptome sequencing was performed by the Metware company (Wuhan, China) based on the Illumina Novoseq 6000 system. The RNA-seq library was constructed using Illumina Stranded Total RNA Prep (3 bioreplicates at a time). The original data were filtered according to the standard to obtain high-quality sequencing data (Clean reads), and then Hisat2 (v2.2.1) was used to locate clean reads and the Atlantic reference genome [81]. A total of 114,021 read counts of genes were standardized by FPKM (Table S10). Differential genes were identified using the default parameter of the DESeq2 (v1.38.0) package. DEG was identified with the truncation value |log2(fold change)| ≥ 2. *p* < 0.05 was used as the screening criteria [82]. FPKM values of StUSP genes were extracted to show the expression patterns of StUSP genes at different processing stages, and heat maps were generated using the Pheatmap (v1.0.12) software package [83]. K-means algorithm was used to perform a cluster analysis of differential genes to screen candidate genes [84]. Unigenes were then queried for the KEGG [85] and Gene Ontology (GO) [86] to obtain functional annotations. The selected differential genes were analyzed via GO annotation and KEGG enrichment with TBtools software.

### 4.8. Weighted Gene Coexpression Network Analysis (WGCNA)

The gene coexpression network was constructed using R software (v4.2.1) and WGCNA (v1.71) [87]. The Pearson correlation coefficient was used to construct the inter-gene correlation matrix (the threshold was 0.8). After threshold filtering, the power adjacency function was used to transform the correlation matrix into an adjacency matrix. In order to characterize the nonlinear relationship between genes, a topological overlap matrix (TOM matrix) was constructed from TOM correlation coefficients. The blockwise modules function of the R software package was used to divide modules and combine similar modules into module eigenvectors (ME matrix) and eigenmatrixes. A coexpression network was constructed to analyze the correlation between module feature genes and central genes. Network visualization was accomplished by computing the central gene via Cytoscape software (v3.7.2) [88].

### 4.9. Real-Time Quantitative PCR (qRT-PCR) Verification

BIOER’s LineGene 9620 real-time fluorescence quantitative PCR system was used to select 10 genes for qRT-PCR analysis, and β-action was used as the reference gene (Table S11). These genes included *StUSP2*, *StUSP6*, *StUSP13*, *StUSP14*, *StUSP15*, *StUSP21*, *StUSP24*, *StUSP9*, *StUSP33*, and *StUSP41*; the primer sequences were designed using prime-blast [89]. Total RNA was extracted from each treated sample using the Total RNA Rapid Extraction Kit (ER501-01, TransGen Biotech, Beijing, China) and reverse-transcribed to cDNA using TransScript^®^ One-Step gDNA Removal and cDNA Synthesis SuperMix (AT311, TransGen Biotech, Beijing, China). qRT-PCR was performed using the ChamQ Universal SYBR qPCR Master Mix (Q711, Vazyme, Nanjing, China) kit. Three biological replicates were calculated using the 2^−∆∆Ct^ method [90].

## 5. Conclusions

In this study, 108 universal stress protein genes were identified from the recently published Atlantic Potato Genome Database. A total of 4 pairs of gene StUSPs were found to be derived from tandem repeats, and 202 pairs of StUSP genes were derived from segmental duplication. This shows that segmental duplication events are the main driving force for the evolution of the potato StUSP gene family. RNA-seq data, WGCNA, and qRT-PCR analysis showed that *StUSP13*, *StUSP14*, *StUSP15*, and *StUSP41* exhibit similar response patterns under multiple stresses, and their corresponding gene networks are related to a variety of signal transductions and biological processes in secondary metabolite synthesis. This research provides a new understanding of the structure, evolution, and function of the plant USP gene family, as well as valuable information for further research on the biological role of the StUSPs gene when potatoes are under adverse conditions.

## Data Availability

The RNA-seq datasets in this study are available from the NCBl Sequence Read Archive under project PRINA943451 and CNCB Sequence Read Archive under project PRJCA021942.

## Supplementary Materials

The following supporting information can be downloaded at: https://www.mdpi.com/article/10.3390/ijms25021341/s1.
