# Supplementary material for: Genome-Wide Identification and Expression Profiling of Potato (Solanum tuberosum L.) Universal Stress Proteins Reveal Essential Roles in Mechanical Damage and Deoxynivalenol Stress
Source: Int J Mol Sci. 2024 Jan 22;25(2):1341. doi: 10.3390/ijms25021341 (PMC10816615; doi:10.3390/ijms25021341)

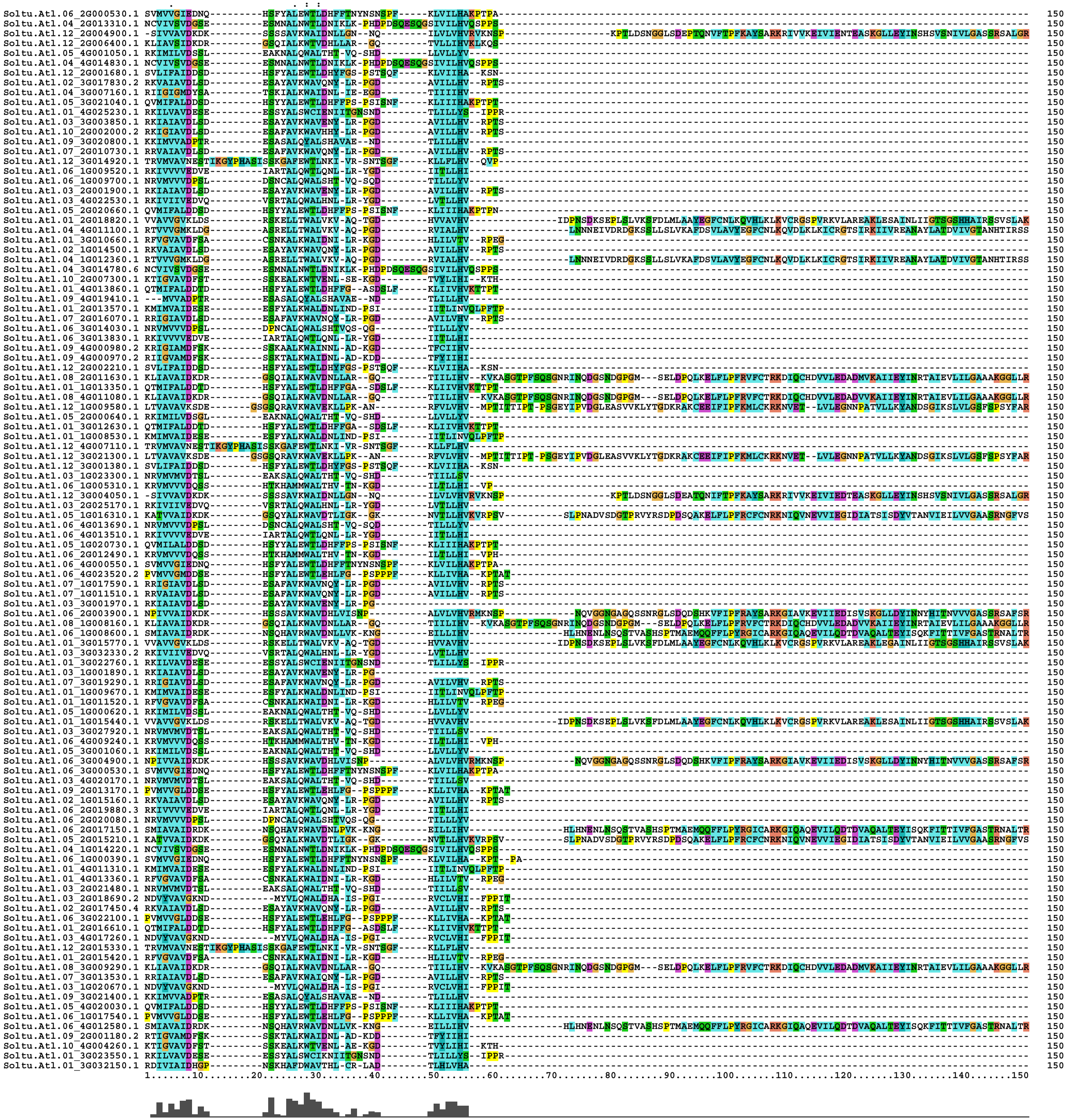

```
Soltu.Atl.06_2G000530.1 ------------------------------------------------------------------------------------------------------------------------------------------------------ 300
Soltu.Atl.04_2G013310.1 ------------------------------------------------------------------------------------------------------------------------------------------------------ 300
Soltu.Atl.12_2G004900.1 KYWTHDVPTLVNKAAPEFCAVYVVSKGKQQSVRPAAKSLPSSVPARQPSSSAWSAPRSSNSGSEDST-RFSYTRSEQKIVESEKTRGEKGPSIVPIDNLHSPEMTNPYKHASQPDGNMRYARINPRSVDMTSENLDFTQVAIRETPMNGS 300
Soltu.Atl.12_2G006400.1 ------------------------------------------------------------------------------------------------------------------------------------------------------ 300
Soltu.Atl.05_4G001050.1 ------------------------------------------------------------------------------------------------------------------------------------------------------ 300
Soltu.Atl.04_4G014830.1 ------------------------------------------------------------------------------------------------------------------------------------------------------ 300
Soltu.Atl.12_2G001680.1 ------------------------------------------------------------------------------------------------------------------------------------------------------ 300
Soltu.Atl.02_3G017830.2 ------------------------------------------------------------------------------------------------------------------------------------------------------ 300
Soltu.Atl.04_3G007160.1 ------------------------------------------------------------------------------------------------------------------------------------------------------ 300
Soltu.Atl.05_3G021040.1 ------------------------------------------------------------------------------------------------------------------------------------------------------ 300
Soltu.Atl.01_4G025230.1 ------------------------------------------------------------------------------------------------------------------------------------------------------ 300
Soltu.Atl.03_3G003850.1 ------------------------------------------------------------------------------------------------------------------------------------------------------ 300
Soltu.Atl.10_2G002000.2 ------------------------------------------------------------------------------------------------------------------------------------------------------ 300
Soltu.Atl.09_3G020800.1 ---------------------------------------------------------------------------------------------------------------------------------G-------------------- 300
Soltu.Atl.07_2G010730.1 ------------------------------------------------------------------------------------------------------------------------------------------------------ 300
Soltu.Atl.12_3G014920.1 ------------------------------------------------------------------------------------------------------------------------------------------------------ 300
Soltu.Atl.06_1G009520.1 ------------------------------------------------------------------------------------------------------------------------------------------------------ 300
Soltu.Atl.06_1G009700.1 ------------------------------------------------------------------------------------------------------------------------------------------------------ 300
Soltu.Atl.03_2G001900.1 ------------------------------------------------------------------------------------------------------------------------------------------------------ 300
Soltu.Atl.03_4G022530.1 ------------------------------------------------------------------------------------------------------------------------------------------------------ 300
Soltu.Atl.05_2G020660.1 ------------------------------------------------------------------------------------------------------------------------------------------------------ 300
Soltu.Atl.01_2G018820.1 YCARKVTKSISVIAVDNGKIVYQREASALDGDESSDSD--------------------------------------------------------------------VPKSRFKRRKTLNKSPLSLMPKKAVEENSCTTENNYMALV---- 300
Soltu.Atl.04_4G011100.1 ----ASVAKYCARKLPKDCSVLAVNNGKVVFQREASLASYASSKELEHHHGNRLLSVIQRTLTKNSKVLNDSTGLRPTNSCREGGYQTLGEALLKAASASAENSLRQNCSVCSPNCLLPDNSCTQTDEEPSDNIHDDYSMAIVPVQSQES 300
Soltu.Atl.01_3G010660.1 ------------------------------------------------------------------------------------------------------------------------------------------------------ 300
Soltu.Atl.02_1G014500.1 ------------------------------------------------------------------------------------------------------------------------------------------------------ 300
Soltu.Atl.04_1G012360.1 ----ASVAKYCARKLPKDCSVLAVNNGKVVFQREASLASYASSKELEHHHGNRLLSVIQRTLTKNSKVLNDSTGLRPTNSCREGGYQTLGEALLKAASASAENSLRQNCSVCSPNCLLPDNSCTQTDEEPSDNNHDDNSMAIVPVQSQES 300
Soltu.Atl.04_3G014780.6 ------------------------------------------------------------------------------------------------------------------------------------------------------ 300
Soltu.Atl.10_2G007300.1 ------------------------------------------------------------------------------------------------------------------------------------------------------ 300
Soltu.Atl.01_4G013860.1 ------------------------------------------------------------------------------------------------------------------------------------------------------ 300
Soltu.Atl.09_4G019410.1 ---------------------------------------------------------------------------------------------------------------------------------G-------------------- 300
Soltu.Atl.01_2G013570.1 ------------------------------------------------------------------------------------------------------------------------------------------------------ 300
Soltu.Atl.07_2G016070.1 ------------------------------------------------------------------------------------------------------------------------------------------------------ 300
Soltu.Atl.06_3G014030.1 ------------------------------------------------------------------------------------------------------------------------------------------------------ 300
Soltu.Atl.06_3G013830.1 ------------------------------------------------------------------------------------------------------------------------------------------------------ 300
Soltu.Atl.09_4G000980.2 ------------------------------------------------------------------------------------------------------------------------------------------------------ 300
Soltu.Atl.09_4G000970.2 ------------------------------------------------------------------------------------------------------------------------------------------------------ 300
Soltu.Atl.12_2G002210.1 ------------------------------------------------------------------------------------------------------------------------------------------------------ 300
Soltu.Atl.08_2G011630.1 -FKIKDIPGNVLKGVPDFCTVYVISKSGKISSTRSAARLAPFIHPLRHQFTQNANVKSNSIEDSNVPPRGSISGIPKPVSDIPMSNLHSDTFNMKSPFTHRKGPNGKPYEISQPDTDISFVSSGRPSIDNMFPSFADTFDSGATPPRLSG 300
Soltu.Atl.01_1G013350.1 ------------------------------------------------------------------------------------------------------------------------------------------------------ 300
Soltu.Atl.08_4G011080.1 -FKIKDIPGNVLKGVPDFCTVYVISKSGKISSTRSAARLAPFIHPLRHQFTQNANVKSNSIEDSNVPPRGSISGIPKPVSDIPMSNLHSDTFNMKSPFTHRKGPNGKPYEISQPDTDISFVSSGRPSIDNMFPSFADTFDSGATPPRLSG 300
Soltu.Atl.12_1G009580.1 KLKGSSVPSIILKHAPDCCDVYVVSSNKLMTNSLNPLLAAEGDLRTINKQQSSASSVSAGSVFHNRSSSVAASHLNSLEFVHGNSSSYVSPQHRTNRNLEDVTTGLEAIKGCHSSTYS-------------------------------- 300
Soltu.Atl.05_2G000640.1 ------------------------------------------------------------------------------------------------------------------------------------------------------ 300
Soltu.Atl.01_3G012630.1 ------------------------------------------------------------------------------------------------------------------------------------------------------ 300
Soltu.Atl.01_1G008530.1 ------------------------------------------------------------------------------------------------------------------------------------------------------ 300
Soltu.Atl.12_4G007110.1 ------------------------------------------------------------------------------------------------------------------------------------------------------ 300
Soltu.Atl.12_3G021300.1 KLKGSSVPSIILKHAPDCCDVYVVSSNKLMTNSLNPLLAAEGDLRTINKQQSSASSVSAGSVFHNRSSSVAASHLNSLEFVHGNSSSYVSPQHRTNRNLEDVTTGLEAIKGCHSSTYS-------------------------------- 300
Soltu.Atl.12_3G001380.1 ------------------------------------------------------------------------------------------------------------------------------------------------------ 300
Soltu.Atl.03_1G023300.1 ------------------------------------------------------------------------------------------------------------------------------------------------------ 300
Soltu.Atl.06_1G005310.1 ------------------------------------------------------------------------------------------------------------------------------------------------------ 300
Soltu.Atl.12_3G004050.1 KYWTHDVPTLVNKAAPEFCAVYVVSKGKQQSVRPAAKSLPSSVPARQPSSSAWSAPRSSNSGSEDSTSRFSYTRSEQKIVESEKTRGEKGPSIVPIDNLHSPEMTNPYKHASQPDGNMRYARISPRSVDMTSENLDFTQVAIRETPMNGS 300
Soltu.Atl.03_2G025170.1 ------------------------------------------------------------------------------------------------------------------------------------------------------ 300
Soltu.Atl.05_1G016310.1 RFKTMDVPTAVSKVIPGFCTVYVIAKGKVQSTKNASSPVPSSPTPVQQNHSTSSLGAKLGFAETRYAQISSDTKGSVTDIRSPYASRSSADDLDSIKSPFNRGKGINRSYGDLSVAESDLSFVSSGRPSSTFPISMDSSHDLGLPPRLSN 300
Soltu.Atl.06_4G013690.1 ------------------------------------------------------------------------------------------------------------------------------------------------------ 300
Soltu.Atl.06_4G013510.1 ------------------------------------------------------------------------------------------------------------------------------------------------------ 300
Soltu.Atl.05_1G020730.1 ------------------------------------------------------------------------------------------------------------------------------------------------------ 300
Soltu.Atl.06_2G012490.1 ------------------------------------------------------------------------------------------------------------------------------------------------------ 300
Soltu.Atl.06_4G000550.1 ------------------------------------------------------------------------------------------------------------------------------------------------------ 300
Soltu.Atl.06_4G023520.2 ------------------------------------------------------------------------------------------------------------------------------------------------------ 300
Soltu.Atl.07_1G017590.1 ------------------------------------------------------------------------------------------------------------------------------------------------------ 300
Soltu.Atl.07_1G011510.1 ------------------------------------------------------------------------------------------------------------------------------------------------------ 300
Soltu.Atl.03_3G001970.1 ------------------------------------------------------------------------------------------------------------------------------------------------------ 300
Soltu.Atl.06_2G003900.1 KFWTHDVPTIVNKAAPDYCTVYVISKGKQQSVRPAAKPLASSSARLPSSQPWSAARLSNYSEPEDISRSAYKNSFGPDMMPKSGTSNASIDSLDFYSRDPKNSYTGRSSPSDDTALFAPLSRGSVDVTTQNLDFTQVSVNDNSS------ 300
Soltu.Atl.08_1G008160.1 -FKIKDIPGNVLKGVPDFCTVYVISKSGKISSTRSAARLAPFIHPLRHQFTQNANVKSNSIEDSNVPPRGSISGIPKPVSDIPMSNLHSDTFNMKSPFTHRKGPNGKPYEISLPDTDISFVSSGRPSIDNMFPSFADTFDSGATPPRLSG 300
Soltu.Atl.06_1G008600.1 AFKIQDVPSSLSRSVPEFCSIYAISRGKVL-KLKSASQPATPSSKASSSQTGFSHNSPISRAHIPQGSWGSIGTFEPIDAGSRSISSDSSSASDRHPASQSTSPNYSSASERTPKAWSDSQHSSRNPSPDRSVSTIGEVFNKIQLTRQPG 300
Soltu.Atl.01_3G015770.1 YCARKVTKSISVIAVDNGKIVYQREASALDGDESSDSD--------------------------------------------------------------------VPISRFKRRKTLNKSPLSLMPKKAVEENSCTTENNYMALV---- 300
Soltu.Atl.03_3G032330.2 ------------------------------------------------------------------------------------------------------------------------------------------------------ 300
Soltu.Atl.01_3G022760.1 ------------------------------------------------------------------------------------------------------------------------------------------------------ 300
Soltu.Atl.03_1G001890.1 ------------------------------------------------------------------------------------------------------------------------------------------------------ 300
Soltu.Atl.07_3G019290.1 ------------------------------------------------------------------------------------------------------------------------------------------------------ 300
Soltu.Atl.01_1G009670.1 ------------------------------------------------------------------------------------------------------------------------------------------------------ 300
Soltu.Atl.01_1G011520.1 ------------------------------------------------------------------------------------------------------------------------------------------------------ 300
Soltu.Atl.05_1G000620.1 ------------------------------------------------------------------------------------------------------------------------------------------------------ 300
Soltu.Atl.01_1G015440.1 YCARKVTKSISVIAVDNGKIVYQREASALDGDESSDSD--------------------------------------------------------------------VPKSRFKRRKTLNKSPLSLMPKKAVEENSCTTENNYMALV---- 300
Soltu.Atl.03_3G027920.1 ------------------------------------------------------------------------------------------------------------------------------------------------------ 300
Soltu.Atl.06_4G009240.1 ------------------------------------------------------------------------------------------------------------------------------------------------------ 300
Soltu.Atl.05_3G001060.1 ------------------------------------------------------------------------------------------------------------------------------------------------------ 300
Soltu.Atl.06_3G004900.1 KFWTHDVPTIVNKAAPDYCTVYVISKGKQQSVRPAAKPLASSSARLPSSQPWSAARLSNYSEPEDISRSAYKNSFGPDMMPKSGTSNASIDSLDFYSRDPKNSYTGRSSPSDDTALFAPLSRGSVDVTTQNLDFTQVSVNDNSS------ 300
Soltu.Atl.06_3G000530.1 ------------------------------------------------------------------------------------------------------------------------------------------------------ 300
Soltu.Atl.03_4G020170.1 ------------------------------------------------------------------------------------------------------------------------------------------------------ 300
Soltu.Atl.09_2G013170.1 ------------------------------------------------------------------------------------------------------------------------------------------------------ 300
Soltu.Atl.02_1G015160.1 ------------------------------------------------------------------------------------------------------------------------------------------------------ 300
Soltu.Atl.06_2G019880.3 ------------------------------------------------------------------------------------------------------------------------------------------------------ 300
Soltu.Atl.06_2G020080.1 ------------------------------------------------------------------------------------------------------------------------------------------------------ 300
Soltu.Atl.06_2G017150.1 AFKIQDVPSSLSRSVPEFCSIYAISR-GKVLKLKSASQPATPSSKASSSQTGFSHNSPISRAHIPQGSWGSIGTFESIDAGSRSISSDSSSASDRHPASQSTSPNYSSASERTPKAWSDSQHSSRNPSPDRSVSTIGEVFNKIQLTRQPG 300
Soltu.Atl.05_2G015210.1 RFKTMDVPTAVSKVIPGFCTVYVIAKGKVQSTKNASSPVPSSPTPVQQNHSTSSLGAKLGFADTRYAQISSDTKGSVTDIRSPYASRSSADDLDSIKSPFNRGKGINRSYGDLSVAESDLSFVSSGRPSSTFPISMDSSHDLGLPPRLSN 300
Soltu.Atl.04_1G014220.1 ------------------------------------------------------------------------------------------------------------------------------------------------------ 300
Soltu.Atl.06_1G000390.1 ------------------------------------------------------------------------------------------------------------------------------------------------------ 300
Soltu.Atl.01_4G011310.1 ------------------------------------------------------------------------------------------------------------------------------------------------------ 300
Soltu.Atl.01_4G013360.1 ------------------------------------------------------------------------------------------------------------------------------------------------------ 300
Soltu.Atl.03_2G021480.1 ------------------------------------------------------------------------------------------------------------------------------------------------------ 300
Soltu.Atl.03_2G018690.2 ------------------------------------------------------------------------------------------------------------------------------------------------------ 300
Soltu.Atl.02_2G017450.4 ------------------------------------------------------------------------------------------------------------------------------------------------------ 300
Soltu.Atl.06_3G022100.1 ------------------------------------------------------------------------------------------------------------------------------------------------------ 300
Soltu.Atl.01_2G016610.1 ------------------------------------------------------------------------------------------------------------------------------------------------------ 300
Soltu.Atl.03_4G017260.1 ------------------------------------------------------------------------------------------------------------------------------------------------------ 300
Soltu.Atl.12_2G015330.1 ------------------------------------------------------------------------------------------------------------------------------------------------------ 300
Soltu.Atl.01_2G015420.1 ------------------------------------------------------------------------------------------------------------------------------------------------------ 300
Soltu.Atl.08_3G009290.1 -FKIKDIPGNVLKGVPDFCTVYVISKSGKISSTRSAARLAPFIHPLRHQFTQNANVKSNSIEDSNVPPRGSISGIPKPVSDIPMSNLHSDTFNMKSPFTHRKGPNGKPYEISQPDTDISFVSSGRPSIDNMFPSFADTFDSGATPPRLSG 300
Soltu.Atl.07_3G013530.1 ------------------------------------------------------------------------------------------------------------------------------------------------------ 300
Soltu.Atl.03_1G020670.1 ------------------------------------------------------------------------------------------------------------------------------------------------------ 300
Soltu.Atl.09_3G021400.1 ---------------------------------------------------------------------------------------------------------------------------------G-------------------- 300
Soltu.Atl.05_4G020030.1 ------------------------------------------------------------------------------------------------------------------------------------------------------ 300
Soltu.Atl.06_1G017540.1 ------------------------------------------------------------------------------------------------------------------------------------------------------ 300
Soltu.Atl.06_4G012580.1 AFKIQDVPSSLSRSVPEFCSIYAISRGKVL-KLKSASQPATPSSKASSSQTGFSHNSPISRAHIPQGSWGSIGTFEPIDAGSRSISSDSSSASDRHPASQSTSPNYSSASERTPKAWSDSQHSSRNPSPDRSVSTIGEVFNKIQLTRQPG 300
Soltu.Atl.09_2G001180.2 ------------------------------------------------------------------------------------------------------------------------------------------------------ 300
Soltu.Atl.10_4G004260.1 ------------------------------------------------------------------------------------------------------------------------------------------------------ 300
Soltu.Atl.01_3G023550.1 ------------------------------------------------------------------------------------------------------------------------------------------------------ 300
Soltu.Atl.01_3G032150.1 ------------------------------------------------------------------------------------------------------------------------------------------------------ 300
                        .......160.......170.......180.......190.......200.......210.......220.......230.......240.......250.......260.......270.......280.......290.......300
```

```
Soltu.Atl.06_2G000530.1 -----------------------------------------------------------------------------------------------------------------------SIIGLS------------------------- 450
Soltu.Atl.04_2G013310.1 -----------------------------------------------------------------------------------------------------------------------IAAGLNPGAIPF------------------- 450
Soltu.Atl.12_2G004900.1 -----------------------------------------------------------------------------------------------------------------------SSYSVDAEMKRLRLELRQTMDMYNAACKEAV 450
Soltu.Atl.12_2G006400.1 ------------------------------------------------------------------------------------------------------------------------------------------------------ 450
Soltu.Atl.05_4G001050.1 ------------------------------------------------------------------------------------------------------------------------------------------------------ 450
Soltu.Atl.04_4G014830.1 -----------------------------------------------------------------------------------------------------------------------IAAGLNPGAIPF------------------- 450
Soltu.Atl.12_2G001680.1 -----------------------------------------------------------------------------------------------------------------------AYATCSVGM---------------------- 450
Soltu.Atl.02_3G017830.2 -----------------------------------------------------------------------------------------------------------------------VLYGAD------------------------- 450
Soltu.Atl.04_3G007160.1 -----------------------------------------------------------------------------------------------------------------------VSSKVEPTNKQL------------------- 450
Soltu.Atl.05_3G021040.1 -----------------------------------------------------------------------------------------------------------------------SVVAI-------------------------- 450
Soltu.Atl.01_4G025230.1 -----------------------------------------------------------------------------------------------------------------------AVYST-------------------------- 450
Soltu.Atl.03_3G003850.1 -----------------------------------------------------------------------------------------------------------------------VLYGAD------------------------- 450
Soltu.Atl.10_2G002000.2 -----------------------------------------------------------------------------------------------------------------------VLYGAD------------------------- 450
Soltu.Atl.09_3G020800.1 -----------------------------------------------------------------------------------------------------------------------NPNAWRNLLGALIKKPLPNS----STSTSTS 450
Soltu.Atl.07_2G010730.1 -----------------------------------------------------------------------------------------------------------------------VLYGAD------------------------- 450
Soltu.Atl.12_3G014920.1 -----------------------------------------------------------------------------------------------------------------------DEDG--------------------------- 450
Soltu.Atl.06_1G009520.1 ------------------------------------------------------------------------------------------------------------------------------------------------------ 450
Soltu.Atl.06_1G009700.1 ------------------------------------------------------------------------------------------------------------------------------------------------------ 450
Soltu.Atl.03_2G001900.1 -----------------------------------------------------------------------------------------------------------------------VLYGAD------------------------- 450
Soltu.Atl.03_4G022530.1 ------------------------------------------------------------------------------------------------------------------------------------------------------ 450
Soltu.Atl.05_2G020660.1 -----------------------------------------------------------------------------------------------------------------------SVVAI-------------------------- 450
Soltu.Atl.01_2G018820.1 -----------------------------------------------------------------------------------------------------------------------PVKPIEVRESKSRWTLLRRVFLQNIVAPDKF 450
Soltu.Atl.04_4G011100.1 GSSSI--------------------------------------------------------------------------------------------------------------TLLIKDLPEVRPGWPLLHRAILSNRQTADTL 450
Soltu.Atl.01_3G010660.1 -----------------------------------------------------------------------------------------------------------------------HYEEGEMQL---------------------- 450
Soltu.Atl.02_1G014500.1 -----------------------------------------------------------------------------------------------------------------------VLYGAD------------------------- 450
Soltu.Atl.04_1G012360.1 GSSSL--------------------------------------------------------------------------------------------------------------TLLIKDLPEVRPGWPLLHRAILSNRQTADTL 450
Soltu.Atl.04_3G014780.6 -----------------------------------------------------------------------------------------------------------------------IAAGLNPGGIPF------------------- 450
Soltu.Atl.10_2G007300.1 -----------------------------------------------------------------------------------------------------------------------TLSESRDQL---------------------- 450
Soltu.Atl.01_4G013860.1 -----------------------------------------------------------------------------------------------------------------------SVVGL-------------------------- 450
Soltu.Atl.09_4G019410.1 -----------------------------------------------------------------------------------------------------------------------NPNAWRNLLGALIKKPLPNS--------STS 450
Soltu.Atl.01_2G013570.1 -----------------------------------------------------------------------------------------------------------------------MVYP--------------------------- 450
Soltu.Atl.07_2G016070.1 -----------------------------------------------------------------------------------------------------------------------VLYGAD------------------------- 450
Soltu.Atl.06_3G014030.1 ------------------------------------------------------------------------------------------------------------------------------------------------------ 450
Soltu.Atl.06_3G013830.1 ------------------------------------------------------------------------------------------------------------------------------------------------------ 450
Soltu.Atl.09_4G000980.2 -----------------------------------------------------------------------------------------------------------------------KNYDSRHQL---------------------- 450
Soltu.Atl.09_4G000970.2 -----------------------------------------------------------------------------------------------------------------------KTHSSDESRNKL------------------- 450
Soltu.Atl.12_2G002210.1 -----------------------------------------------------------------------------------------------------------------------AYATCSVGM---------------------- 450
Soltu.Atl.08_2G011630.1 FSDYEGQSFEPMPMGRRSLDIMPSEMSFLSMEGDRPSFSS-------------------------------------------------------------------------------TPDDVAAEMRRLKLELKQTMEMYSTACKEAL 450
Soltu.Atl.01_1G013350.1 -----------------------------------------------------------------------------------------------------------------------SVVGL-------------------------- 450
Soltu.Atl.08_4G011080.1 FSDYEGQSFEPMPMGRRSLDIMPSEMSFLSMEGDRPSFSS-------------------------------------------------------------------------------TPDDVAAEMRRLKLELKQTMEMYSTACKEAL 450
Soltu.Atl.12_1G009580.1 -----------------------------------------------------------------------------------------------------------------------EQLDIQDEVERLHLELQDTLAMYNQACEDLT 450
Soltu.Atl.05_2G000640.1 ------------------------------------------------------------------------------------------------------------------------------------------------------ 450
Soltu.Atl.01_3G012630.1 -----------------------------------------------------------------------------------------------------------------------SVVGL-------------------------- 450
Soltu.Atl.01_1G008530.1 -----------------------------------------------------------------------------------------------------------------------MVYP--------------------------- 450
Soltu.Atl.12_4G007110.1 -----------------------------------------------------------------------------------------------------------------------QVPDEDEEI-----------AMGDSNTIPIS 450
Soltu.Atl.12_3G021300.1 -----------------------------------------------------------------------------------------------------------------------EQLDIQDEVERLHLELQDTLAMYNQACEDLT 450
Soltu.Atl.12_3G001380.1 -----------------------------------------------------------------------------------------------------------------------AYATCSVGM---------------------- 450
Soltu.Atl.03_1G023300.1 ------------------------------------------------------------------------------------------------------------------------------------------------------ 450
Soltu.Atl.06_1G005310.1 ------------------------------------------------------------------------------------------------------------------------------------------------------ 450
Soltu.Atl.12_3G004050.1 -----------------------------------------------------------------------------------------------------------------------SSYSVDAEMKRLRLELRQTMDMYNAACKEAV 450
Soltu.Atl.03_2G025170.1 ------------------------------------------------------------------------------------------------------------------------------------------------------ 450
Soltu.Atl.05_1G016310.1 SSDTDVKYSAPRLSNSSETESRLSFGSSFSATRLSEANGFSSNSFDSGNGSWSSPS---------------------------------------------------------------NMEDIEAEMRRLKQELKQTMDMYSSACKEAL 450
Soltu.Atl.06_4G013690.1 ------------------------------------------------------------------------------------------------------------------------------------------------------ 450
Soltu.Atl.06_4G013510.1 ------------------------------------------------------------------------------------------------------------------------------------------------------ 450
Soltu.Atl.05_1G020730.1 -----------------------------------------------------------------------------------------------------------------------SVVAI-------------------------- 450
Soltu.Atl.06_2G012490.1 ------------------------------------------------------------------------------------------------------------------------------------------------------ 450
Soltu.Atl.06_4G000550.1 -----------------------------------------------------------------------------------------------------------------------SIIGLA------------------------- 450
Soltu.Atl.06_4G023520.2 -----------------------------------------------------------------------------------------------------------------------SAIGL-------------------------- 450
Soltu.Atl.07_1G017590.1 -----------------------------------------------------------------------------------------------------------------------VLYGAD------------------------- 450
Soltu.Atl.07_1G011510.1 -----------------------------------------------------------------------------------------------------------------------VLYGAD------------------------- 450
Soltu.Atl.03_3G001970.1 ------------------------------------------------------------------------------------------------------------------------------------------------------ 450
Soltu.Atl.06_2G003900.1 -----------------------------------------------------------------------------------------------------------------------SSSMWESEMSRLKLELKQTMDMYNSACKDAV 450
Soltu.Atl.08_1G008160.1 FSDYEGQSFEPMPMGRRSLDIMPSEMSFLSMEGDRPSFSS-------------------------------------------------------------------------------TPDDVAAEMRRLKLELKQTMEMYSTACKEAL 450
Soltu.Atl.06_1G008600.1 CKNLTPIPSWGNSGDFGEWAPPAELVGSSENSSQTFMNSNMDTTKILHDRHLGSLLPPSQHKVNNLNLRMRPKENSFYSTSGSSNLSSSSSYRFSDMSFEHSDSSHNVSDASRCSISSQNADELEEEMKRLKFELKQSLDMHNGIC---- 450
Soltu.Atl.01_3G015770.1 -----------------------------------------------------------------------------------------------------------------------PVKPIEVRESKSRWTLLRRVFLQNIVAPDKF 450
Soltu.Atl.03_3G032330.2 ------------------------------------------------------------------------------------------------------------------------------------------------------ 450
Soltu.Atl.01_3G022760.1 -----------------------------------------------------------------------------------------------------------------------AVYST-------------------------- 450
Soltu.Atl.03_1G001890.1 ------------------------------------------------------------------------------------------------------------------------------------------------------ 450
Soltu.Atl.07_3G019290.1 -----------------------------------------------------------------------------------------------------------------------VLYGAD------------------------- 450
Soltu.Atl.01_1G009670.1 -----------------------------------------------------------------------------------------------------------------------MVYP--------------------------- 450
Soltu.Atl.01_1G011520.1 -----------------------------------------------------------------------------------------------------------------------HYEEGEMQL---------------------- 450
Soltu.Atl.05_1G000620.1 ------------------------------------------------------------------------------------------------------------------------------------------------------ 450
Soltu.Atl.01_1G015440.1 -----------------------------------------------------------------------------------------------------------------------PVKPIEVRESKSRWTLLRRVFLQNIVAPDKF 450
Soltu.Atl.03_3G027920.1 ------------------------------------------------------------------------------------------------------------------------------------------------------ 450
Soltu.Atl.06_4G009240.1 ------------------------------------------------------------------------------------------------------------------------------------------------------ 450
Soltu.Atl.05_3G001060.1 ------------------------------------------------------------------------------------------------------------------------------------------------------ 450
Soltu.Atl.06_3G004900.1 -----------------------------------------------------------------------------------------------------------------------SSSMWESEMSRLKLELKQTMDMYNSACKDAV 450
Soltu.Atl.06_3G000530.1 -----------------------------------------------------------------------------------------------------------------------SIIGLA------------------------- 450
Soltu.Atl.03_4G020170.1 ------------------------------------------------------------------------------------------------------------------------------------------------------ 450
Soltu.Atl.09_2G013170.1 -----------------------------------------------------------------------------------------------------------------------SAIGL-------------------------- 450
Soltu.Atl.02_1G015160.1 -----------------------------------------------------------------------------------------------------------------------VLYGAD------------------------- 450
Soltu.Atl.06_2G019880.3 ------------------------------------------------------------------------------------------------------------------------------------------------------ 450
Soltu.Atl.06_2G020080.1 ------------------------------------------------------------------------------------------------------------------------------------------------------ 450
Soltu.Atl.06_2G017150.1 CKNLTPIPSWSNSGDFGEWAPPAELVGSSENSSQAFMTSNMDTKKILHDRHLGSLLPPSQHKVNNLNLRMRPKENSFYSTSGSSNLSSSSSYRFSDMSFEHSDSSHNVSDASRCSISSQNADELEEEMKRLKFELKQSLDMHNGICR--- 450
Soltu.Atl.05_2G015210.1 SSDTDVKYSAPRLSNSSETESRLSFGSSFSATRLSEANGFSSNSFDSGNGSWSSPS---------------------------------------------------------------NMEDIEAEMRRLKQELKQTMDMYSSACKEAL 450
Soltu.Atl.04_1G014220.1 -----------------------------------------------------------------------------------------------------------------------IAAGLNPGAIPF------------------- 450
Soltu.Atl.06_1G000390.1 -----------------------------------------------------------------------------------------------------------------------SIIGLAAGP---------------------- 450
Soltu.Atl.01_4G011310.1 -----------------------------------------------------------------------------------------------------------------------MVYP--------------------------- 450
Soltu.Atl.01_4G013360.1 -----------------------------------------------------------------------------------------------------------------------HYEEGEMQL---------------------- 450
Soltu.Atl.03_2G021480.1 ------------------------------------------------------------------------------------------------------------------------------------------------------ 450
Soltu.Atl.03_2G018690.2 ------------------------------------------------------------------------------------------------------------------------------------------------------ 450
Soltu.Atl.02_2G017450.4 -----------------------------------------------------------------------------------------------------------------------VLYGAD------------------------- 450
Soltu.Atl.06_3G022100.1 -----------------------------------------------------------------------------------------------------------------------SAIGL-------------------------- 450
Soltu.Atl.01_2G016610.1 -----------------------------------------------------------------------------------------------------------------------SVVGL-------------------------- 450
Soltu.Atl.03_4G017260.1 ------------------------------------------------------------------------------------------------------------------------------------------------------ 450
Soltu.Atl.12_2G015330.1 -----------------------------------------------------------------------------------------------------------------------QVPDEDG------------------------ 450
Soltu.Atl.01_2G015420.1 -----------------------------------------------------------------------------------------------------------------------HYEEGEMQL---------------------- 450
Soltu.Atl.08_3G009290.1 FSDYEGQSFEPMPMGRRSLDIMPSEMSFLSMEGDRPSFSS-------------------------------------------------------------------------------TPDDVAAEMRRLKLELKQTMEMYSTACKEAL 450
Soltu.Atl.07_3G013530.1 -----------------------------------------------------------------------------------------------------------------------VLYGAD------------------------- 450
Soltu.Atl.03_1G020670.1 ------------------------------------------------------------------------------------------------------------------------------------------------------ 450
Soltu.Atl.09_3G021400.1 -----------------------------------------------------------------------------------------------------------------------NPNAWRNLLGALIKKPLPNSSMSTSTSTSTS 450
Soltu.Atl.05_4G020030.1 -----------------------------------------------------------------------------------------------------------------------SVVAI-------------------------- 450
Soltu.Atl.06_1G017540.1 -----------------------------------------------------------------------------------------------------------------------SAIGL-------------------------- 450
Soltu.Atl.06_4G012580.1 CKNLTPIPSWGNSGDFGEWAPPAELVGSSENSSQTFMNSNMDTTKILHDRHLGSLLPPSQHKVNNLNLRMRPKENSFYSTSGSSNLSSSSSYRFSDMSFEHSDSSHNVSDASRCSISSQNADELEEEMKRLKFELKQSLDMHNGIC---- 450
Soltu.Atl.09_2G001180.2 -----------------------------------------------------------------------------------------------------------------------KTHSSDESRNKL------------------- 450
Soltu.Atl.10_4G004260.1 -----------------------------------------------------------------------------------------------------------------------TLSESRDQL---------------------- 450
Soltu.Atl.01_3G023550.1 -----------------------------------------------------------------------------------------------------------------------AVYSTLDG----------------------- 450
Soltu.Atl.01_3G032150.1 ------------------------------------------------------------------------------------------------------------------------------------------------------ 450
                        ......310.......320.......330.......340.......350.......360.......370.......380.......390.......400.......410.......420.......430.......440.......450
```

```
Soltu.Atl.06_2G000530.1 ----------------------AGPG---SVEAFSAIESNLK-----------------------------------------------------KIAARVVEKAMEICKAKSVN---------------DA--VVEIVEG---DARH--   600
Soltu.Atl.04_2G013310.1 ----------------------GGPS---DIEVPAFTAAIEAHQK--------------------------------------------------RITQAILDHALEICANKNAN----------------V--KTQVVIG---DPKE--   600
Soltu.Atl.12_2G004900.1 VANQAADELNKWKSEEARKFEQARFA---EEAALAIAETEKAKCRAAIEAAKKAQKMAEIEAQRRKYAELKAKRETEKKKQAMNVRSQNDIRYRKYTIEEIEAATKKFSNAQKIGEGGYGPVFKGKLDHTPV--AIKVLSADAAQGKK--   600
Soltu.Atl.12_2G006400.1 ----------------------ANAS---GQSTSSKSNQNSDEGGSGNIELD-------------------------------------------QQTKELFLPFRAFCTRKDI----------------QC--YVIVLDD--TDVAR--   600
Soltu.Atl.05_4G001050.1 ------------------------------TKPPSSSSSFSKQDEDSKKEIN-------------------------------------------PRIFEFLASMKNICKMKRPE--------------IQV--EVAVAQG--KEKGP--   600
Soltu.Atl.04_4G014830.1 ----------------------GGPS---DIEVPAFTAAIEAHQK--------------------------------------------------RITQAILDHALEICANKNAN----------------V--KTQVVIG---DPKE--   600
Soltu.Atl.12_2G001680.1 ----------------------SGPG---RIDVLTLIETDIK-----------------------------------------------------RAVDKVIEKAKELCKSKGVT---------------NV--SYEVYEG---DARN--   600
Soltu.Atl.02_3G017830.2 ----------------------WGAI---DVSVDTENEESQRKLEDDFDN---------------------------------------------FTTTKSNDLAQPLVEASIPF-------------------KIHIVKD--HDMKE--   600
Soltu.Atl.04_3G007160.1 ----------------------FEDT---GSPLIPLDEFKQINVSKYYGL---------------------------------------------NPDKEVIDMLEPVAKLKKVT----------------V--VSKVYWG---DARE--   600
Soltu.Atl.05_3G021040.1 ----------------------AGPG---TSDMFTLVETDIT-----------------------------------------------------KAAQKTIDKSKELCKTKGVS---------------NV--ACEILEG---DARN--   600
Soltu.Atl.01_4G025230.1 -----------------------------LDGTGYLFSSDILATMERYSS---------------------------------------------EVAQCVMEKAKRACEALNGV---------------KV--ETIVEHG---DARD--   600
Soltu.Atl.03_3G003850.1 ----------------------WGAI---DVSIDTTDEKSKQKLEEDFDN---------------------------------------------FTSTKSNDLAQPLVDANIPF-------------------KIRIVKD--HDMKE--   600
Soltu.Atl.10_2G002000.2 ----------------------WGSV---DLSIVDTDNIESQKKLEDDFDA--------------------------------------------FTSAKSADLAQPLVEVQIPY-------------------KIHIVKD--HDMKE--   600
Soltu.Atl.09_3G020800.1 TSPSDSSPFSTFFR--------KSLT---NTTSPQSTSAASCEAAEDGGDVK-----GCTG----------------------------------KGYMNFLGTMKHACEVAQPK--------------IKVCTETVEMDGNAKEKAT--   600
Soltu.Atl.07_2G010730.1 ----------------------WGSA----------STSPAHKIERDYEI---------------------------------------------FTNNKATELALPLVEANVPF-------------------KIHIVKD--RDMKE--   600
Soltu.Atl.12_3G014920.1 ----------------------FEDM---DSIFASPDDFKGMKNRDKIRGLH------------------------------------------------LLEYFVNRCHEIGV----------------PC--EAWIKKG---DPKE--   600
Soltu.Atl.06_1G009520.1 ------------------------------------FSITRSKSKKKLRLLR-------------------------------------------LQGFQLALSFQDICNNFFNT---------------KT--EIVVTQG--DQDGG--   600
Soltu.Atl.06_1G009700.1 -------------------------------------SKISKEGEKANSEIN-------------------------------------------QRAYELLCSMKNMCQTKKPG--------------VQV--EIVMQEG--KEKGA--   600
Soltu.Atl.03_2G001900.1 ----------------------WGAI---DVSIDTTDEKSKQKLEEDFDN---------------------------------------------FTSTKSNDLAQPLVDANIPF-------------------KIRIVKD--HDMKE--   600
Soltu.Atl.03_4G022530.1 ------------------------------------FPNLRSRTSNKLRLLR-------------------------------------------LKGFQLALSFQDLCNNFPNT-------------------KTEIVVTEEDQDGG--   600
Soltu.Atl.05_2G020660.1 ----------------------AGPG---TSDMFTLVETDIT-----------------------------------------------------KAAQKTIDKSKELCKTKGVS---------------NV--ACEILEG---DARN--   600
Soltu.Atl.01_2G018820.1 PAKRSSVMHWVWKRPSRQSFAAIYPD---HKQSVSDKDEPHRTNLDEGKGAI-----IPVGSDADPISNECFIVLP-------------------EELEGLTERYSSMCRLFNYQELCSATSDFLPENLIGKGGSSKVYKGCLPDGKQLA   600
Soltu.Atl.04_4G011100.1 SIRKLSVVQWALCLPTRHLLCIEDAD---RRDLHSADAERLAPALDEKSGAIVPVNHETTSSKSSPENSPRALP---------------------RELDGLHEKYSATCRLFKFQELLLATLNFSSESIIGKGGSSQVFKGCLPDGKELA   600
Soltu.Atl.01_3G010660.1 ----------------------WEAT---GSPLIPLSEICDGHTMKRYGV---------------------------------------------NPDPETLQMITLAARQMEIT----------------V--VMKIFWG---DARE--   600
Soltu.Atl.02_1G014500.1 ----------------------WGAI---DVSVDTENEESQRKLEDDFDN---------------------------------------------FTTTKSNDLAQPLVEANIPF-------------------KIHIVKD--HDMKE--   600
Soltu.Atl.04_1G012360.1 SIRKLSVVQWALCLPTRHLLCIEDAD---RRDLHSADAERLAPALDEKSGAIVPVNHETTSSKSSPENSPRALP---------------------RELDGLHEKYSATCRLFKFQELLLATLNFSSESIIGKGGSSQVFKGCLPDGKELA   600
Soltu.Atl.04_3G014780.6 ----------------------GGPS---DIEVPAFTAAIEAHQK--------------------------------------------------RITQAILDHALEICANKNAN----------------V--KTQVVIG---DPKE--   600
Soltu.Atl.10_2G007300.1 ----------------------WSKT---GSPLIPWVEFRDPEVMKYYDIK--------------------------------------------KTDVQVVDLLDAATKQKEIN----------------I--VAKVYWG---DARE--   600
Soltu.Atl.01_4G013860.1 ----------------------AGPG---TSDVLVVVEKDIK-----------------------------------------------------KTAQRVCEKSKQLCEAKGVN---------------GV--EFDVVEG---DARN--   600
Soltu.Atl.09_4G019410.1 TSPSDSSPFSTFFR--------KSLT---NTTSPQPTSAASHAAAEDGGDVK-----GCTG----------------------------------KGYMNFLGTMKHACEVAQPK--------------IKVCTETVEMDGNAKEKAT--   600
Soltu.Atl.01_2G013570.1 ----------------------AGPV---VFAAPTVVEAVKKGQH--------------------------------------------------ENATRILSRALHLCKQKMVKA------------------ETLILEG---DPKD--   600
Soltu.Atl.07_2G016070.1 ----------------------WGSV---DLSIVDTENEESQRKLEDEFDT--------------------------------------------FTTTKASDLAQPLVDAQIPF-------------------KIHIVKD--HDMRE--   600
Soltu.Atl.06_3G014030.1 -------------------------------------SKISKEGEKANIEIN-------------------------------------------QRAYELLCSMKNMCQTKKPG--------------VQV--EIVMQEG--KEKGA--   600
Soltu.Atl.06_3G013830.1 ------------------------------------FSITRSKSKKKLRLLR-------------------------------------------LQGFQLALSFQDICNNFFNT---------------KT--EIVVTQG--DQDGG--   600
Soltu.Atl.09_4G000980.2 ----------------------WAQS---GSPLIPLVEFREAEVLRYYDV---------------------------------------------ETDIELLDMLDTATTQKQIN----------------V--VIKLYWG---DARE--   600
Soltu.Atl.09_4G000970.2 ----------------------WAQS---GSPLIPLAEFREPEVLEKYGV---------------------------------------------EIDIELLDMLDTASRQKEIK----------------V--VTKLYWG---DARE--   600
Soltu.Atl.12_2G002210.1 ----------------------SGPG---RIDVLTLIETDIK-----------------------------------------------------RAVDKVIEKAKELCKSKGVT---------------NV--SYEVYEG---DARN--   600
Soltu.Atl.08_2G011630.1 TAQQKATELQRWKAEEQRRIEEARFA---EEAALALAEKEKAKSRAALEHAEAAQRLAELESQKRINAEMKALKEAEERNKVLNKLSNSDFRYRRYSIEEIESATNYFAQSNKIGEGGYGPVYKCYMDHTHV--AVKVLRPDAAHGRQ--   600
Soltu.Atl.01_1G013350.1 ----------------------AGPG---TSDVLVVVEKDIK-----------------------------------------------------KTAQRVCEKSKQLCEAKGVN---------------GV--EFDVVEG---DARN--   600
Soltu.Atl.08_4G011080.1 TAQQKATELQRWKAEEQRRIEEARCA---EEAALALAEKEKAKSRAALEHAEAAQRLAELESQKRINAEMKALKEAEERNKVLNKLSNSDFRYRRYSIEEIESATNYFAQSNKIGEGGYGPVYKCYMDHTHV--AVKVLRPDAAHGRQ--   600
Soltu.Atl.12_1G009580.1 HARNKVQLFSSQYLEESGKVNAAKKR---EENLRKIAAEEKEKHMEAEKEVEIARKLLSKEVYERQIAELKALQQSLEKKRIVDALLSSDGRYRRFTRGEIEVATDYFSESKMIGEGAYGKVYKGDLEHTPV--AIKVLHSDASEKKE--   600
Soltu.Atl.05_2G000640.1 ----------------------------------TKPSSSSKQDEDSKKEIN-------------------------------------------PRIFAFLASMKNICKMKRPE--------------IQV--EVAVAQG--KEKGP--   600
Soltu.Atl.01_3G012630.1 ----------------------AGPG---TSDVLVVVEKDIK-----------------------------------------------------KTAQRVCEKSKQLCEAKGVN---------------GV--EFDVVEG---DARN--   600
Soltu.Atl.01_1G008530.1 ----------------------AGPV---VFAAPTVVEAVKKGQH--------------------------------------------------ENATRILSRALHLCKQKMV----------------KA--ETLILDG---DPKD--   600
Soltu.Atl.12_4G007110.1 HTGSDFILYXXXXGEAVKPLGYSGRG---KTKFTLLLDRNKKGFEDMDSIFASPDDFKGMKNRDK------------------------------IRGLHLLEYFVNRCHEIGVP----------------C--EAWIKKG---DPKE--   600
Soltu.Atl.12_3G021300.1 HARNKVQLFSSQYLEESGKVNAAKKR---EENLRKIAAEEKEKHMEAEKEVEIARKLLSKEVYERQIAELKALQQSLEKKRIVDALLSSDGRYRRFTRGEIEVATDYFSESKMIGEGAYGKVYKGDLEHTPV--AIKVLHSDASEKKE--   600
Soltu.Atl.12_3G001380.1 ----------------------SGPG---RIDVLTLIETDIK-----------------------------------------------------RAVDKVIEKAKELCKSKGVT---------------NV--SYEVYEG---DARN--   600
Soltu.Atl.03_1G023300.1 -------------------------------------IKPTQRGENENSETN-------------------------------------------QRAYELLYSMKTMCQTKRPE--------------VQV--EIAVEEG--KEKGP--   600
Soltu.Atl.06_1G005310.1 -----------------------------HSSSSSSSHCANNKGFSSDSSS--------------------------------------------SSAAHLASSLGSLCKACKPE--------------VEV--EALVIQG--PKMA---   600
Soltu.Atl.12_3G004050.1 VANQAADELNKWKSEEARKFEQARFA---EEAALAIAETEKAKCRAAIEAAKKAQKMAEIEAQRRKYAELKAKRETEKKKQAMNVRSQNDIRYRKYTIEEIEAATKKFSNAQKIGEGGYGPVFKGKLDHTPV--AIKVLSADAAQGKK--   600
Soltu.Atl.03_2G025170.1 ------------------------------------FPNLRSRTSNKLRLLR-------------------------------------------LKGFQLALSFQDLCNNFPNT---------------KT--EIVVTEE--DQDGG--   600
Soltu.Atl.05_1G016310.1 SAKHKAMELHRWKVEEEQKLEEARLA---EEAALAVAEKEKAKCRAALEAAEEAQRIAEREAQRRISAERKALKESEEKKKVLDALAQSDCRYRKYTIEEIETATDNFAATRKIGEGGYGPVYKCYLDHTQV--AIKVLRPDAAQGRS--   600
Soltu.Atl.06_4G013690.1 -------------------------------------SKISKEGEKANSEIN-------------------------------------------QRAYELLCSMKNMCQTKKPG--------------VQV--EIVMQEG--KEKGA--   600
Soltu.Atl.06_4G013510.1 ------------------------------------FSITRSKSKKKLRLLR-------------------------------------------LQGFQLALSFQDICNNFFNT---------------KT--EIVVTQG--DQDGG--   600
Soltu.Atl.05_1G020730.1 ----------------------AGPG---TSDMFTLVETDIT-----------------------------------------------------KAAQKTIDKSKELCKTKGVS---------------NV--ACEILEG---DARN--   600
Soltu.Atl.06_2G012490.1 ------------------------------SSSSSSSHCANNKGFSSDSSS--------------------------------------------SSAAHLASSLGSLCKACKPE--------------VEV--EALVIQG--PKMA---   600
Soltu.Atl.06_4G000550.1 ----------------------AGPG---SVEAFSAIESNLK-----------------------------------------------------KIAARVVEKAMEICKAKSVN---------------DV--VVEIVEG---DARH--   600
Soltu.Atl.06_4G023520.2 ----------------------AGPG---GADVLPYVEADLR-----------------------------------------------------KISSRIAEKAKKICSAKSVN---------------DV--VVEIVEG---DARS--   600
Soltu.Atl.07_1G017590.1 ----------------------WGSV---DLSIVDTENEESQRKLEDEFDT--------------------------------------------FTTTKASDLAQPLVDAQIPF-------------------KIHIVKD--HDMRE--   600
Soltu.Atl.07_1G011510.1 ----------------------WGST----------STSPAHKIERDYEI---------------------------------------------FTNNKATELALPLVEANVPF-------------------KIHIVKD--RDMKE--   600
Soltu.Atl.03_3G001970.1 ------------------------------------------------------------------------------------------------------------------------------------------------------   600
Soltu.Atl.06_2G003900.1 SANQTAKELHQWKVEEGSRFKQSRIS---EEAALAMAEMEKAKGRAAVEAAQKSQRLAEIEAKRRRYAELKARRETEEKNLAINDLSRNEICYRKYTIEEIEITTKSFSNSEKIGEGGYGPVYKGRLDHTPV--AIKVLRSDAAQGMQ--   600
Soltu.Atl.08_1G008160.1 TAQQKATELQRWKAEEQRRIEEARFA---EEAALALAEKEKAKSRAALEHAEAAQRLAELESQKRINAEMKALKEAEERNKVLNKLSNSDFRYRRYSIEEIESATNYFAQSNKIGEGGYGPVYKCYMDHTHV--AVKVLRPDAAHGRQ--   600
Soltu.Atl.06_1G008600.1 --RRQAREIDRSTSKEACNLEEAKEA---PEASHAMLEKEKQKCKAALEVAQMAQHMAELESKKRKGVEKKFRHEAEEKKKAQDAFAGSENCYRIYSTDEIEAATNYFSNSQKIGEGGYGPVYQGYLDHTSV--AIKVLRSDITQGQI--   600
Soltu.Atl.01_3G015770.1 PAKRSSVMHWVWKRPSRQSFAAIYPD---HKQSVSDKDEPHCTNLDEGKGAI-----IPVGSDADPISNECFIVLP-------------------EELEGLTERYSSMCRLFNYQELCSATSDFLPENLIGKGGSSKVYKGCLPDGKQLA   600
Soltu.Atl.03_3G032330.2 ------------------------------------FPNLRSRTSNKLRLLR-------------------------------------------LKGFQLALSFQDLCNNFPNT---------------KT--EIVVTEE--DQDGG--   600
Soltu.Atl.01_3G022760.1 -----------------------------LDGTGYLFSSDILATMERYSS---------------------------------------------EVAQCVMEKAKRACEALNGV---------------KV--ETIVEHG---DARD--   600
Soltu.Atl.03_1G001890.1 ------------------------------------------------------------------------------------------------------------------------------------------------------   600
Soltu.Atl.07_3G019290.1 ----------------------WGSV---DLSIVDTENEESQRKLEDEFDT--------------------------------------------FTTTKASDLAQPLVDAQIPF-------------------KIHIVKD--HDMRE--   600
Soltu.Atl.01_1G009670.1 ----------------------AGPV---VFAAPTVVEAVKKGQH--------------------------------------------------ENATRILSRALHLCKQKMVKA------------------ETLILDG---DPKD--   600
Soltu.Atl.01_1G011520.1 ----------------------WEAT---GSPLIPLSEICDGHTMKRYGV---------------------------------------------NPDPETLQMITLAARQMEIT----------------V--VMKIFWG---DARE--   600
Soltu.Atl.05_1G000620.1 ----------------------------------TKPSSSSKQDEDSKKEIN-------------------------------------------PRIFEFLASMKNICKMKRPE--------------VQV--EVAVAQG--KEKGP--   600
Soltu.Atl.01_1G015440.1 PAKRSSVMHWVWKRPSRQSFAAIYPD---HKQSVSDKDEPHRTNLDEGKGAI-----IPVGSDADPISNECFIVLP-------------------EELEGLTERYSSMCRLFNYQELCSATSDFLPENLIGKGGSSKVYKGCLPDGKQLA   600
Soltu.Atl.03_3G027920.1 -------------------------------------IKPTQRGENENSETN-------------------------------------------QRAYELLYSMKTMCQTKRPE--------------VQV--EIAVEEG--KEKGP--   600
Soltu.Atl.06_4G009240.1 ------------------------------SSSSSSSHCANNKGFSSDSSS--------------------------------------------SSAAHLASSLGSLCKACKPE--------------VEV--EALVIQG--PKMA---   600
Soltu.Atl.05_3G001060.1 ----------------------------------TKPSSSSKQDEDSKKEIN-------------------------------------------PRIFEFLASMKNICKMKRPE--------------IQV--EVAVAQG--KEKGP--   600
Soltu.Atl.06_3G004900.1 SANQTAKELHQWKVEEGSRFKQSRIS---EEAALAMAEMEKAKGRAAVEAAQKSQRLAEIEAKRRRYAELKARRETEEKNLAINDLSRNEICYRKYTIEEIEITTKSFSNSEKIGEGGYGPVYKGRLDHTPV--AIKVLRSDAAQGMQ--   600
Soltu.Atl.06_3G000530.1 ----------------------AGPG---SVEAFSAIESNLK-----------------------------------------------------KIAARVVEKAMEICKAKSVN---------------DV--VVEMVEG---DARH--   600
Soltu.Atl.03_4G020170.1 -------------------------------------IKPTQRGENENSETN-------------------------------------------QRAYELLYSMKTMCQTKRPE--------------VQV--EIAVEEG--KEKGP--   600
Soltu.Atl.09_2G013170.1 ----------------------AGPG---GADVLPYVEADLR-----------------------------------------------------KISSRIAEKAKKICSAKSVN---------------DV--VVEIVEG---DARS--   600
Soltu.Atl.02_1G015160.1 ----------------------WGAI---DVSVDTENEESQRKLEDDFDN---------------------------------------------FTTTKSNDLAQPLVEANIPF-------------------KIHIVKD--HDMKE--   600
Soltu.Atl.06_2G019880.3 ------------------------------------FSITRSKSKKKLRLLR-------------------------------------------LQGFQLALSFQDICNNFFNT---------------KT--EIVVTQG--DQDGG--   600
Soltu.Atl.06_2G020080.1 -------------------------------------SKISKEGEKANIEIN-------------------------------------------QRAYELLCSMKNMCQTKKPG--------------VQV--EIVMQEG--KEKGA--   600
Soltu.Atl.06_2G017150.1 ---RQAREIDRSTSKEACNLEEAKEA---PEASHAMLEKEKQKCKAALEVAQMAQHMAELESKKRKGVEKKFRHEAEEKKKAQDAFAGSENCYRRYSTDEIEAATNYFSNSQKIGEGGYGPVYKGYLDHTSV--AIKVLRSDITQGQI--   600
Soltu.Atl.05_2G015210.1 SAKHKAMELHRWKVEEEQKLEEARLA---EEAALAVAEKEKAKCRAALEAAEEAQRIAEREAQRRISAERKALKESEEKKKVLDALAQSDCRYRKYTIEEIETATDNFAATRKIGEGGYGPVYKCYLDHTQV--AIKVLRPDAAQGRS--   600
Soltu.Atl.04_1G014220.1 ----------------------GGPS---DIEVPAFTAAIEAHQK--------------------------------------------------RITQAILDHALEICANKNAN----------------V--KTQVVIG---DPKE--   600
Soltu.Atl.06_1G000390.1 -------------------------G---SVEAFSAIESNLK-----------------------------------------------------KIAARVVEKAMEICKAKSVN---------------DV--VVEMVEG---DARH--   600
Soltu.Atl.01_4G011310.1 ----------------------AGPV---VFAAPTVVEAVKKGQH--------------------------------------------------ENATRILSRALHLCKQKMVK--------------A----ETLILDG---DPKD--   600
Soltu.Atl.01_4G013360.1 ----------------------WEAT---GSPLIPLSEICDGHTMKRYGV---------------------------------------------NPDPETLQMITLAARQMEIT----------------V--VMKIFWG---DARE--   600
Soltu.Atl.03_2G021480.1 -------------------------------------IKPTKQGENENSETN-------------------------------------------QRAYELLYSMKTLCQTKRPE--------------VQV--EIAVEEG--KEKGP--   600
Soltu.Atl.03_2G018690.2 ----------------------YIPS---PVGKLSSSQLTREQVQAYINEES-------------------------------------------NRRKNLLEKYIRLCNDAKV----------------PV--DTVLVES--KSPGK--   600
Soltu.Atl.02_2G017450.4 ----------------------WGAI---DVSVDTENEESQRKLEDDFDN---------------------------------------------FTTTKSNDLAQPLVEANIPF-------------------KIHIVKD--HDMKE--   600
Soltu.Atl.06_3G022100.1 ----------------------AGPG---GADVLPYVEADLR-----------------------------------------------------KISSRIAEKAKKICSAKSVN---------------DV--VVEIVEG---DARS--   600
Soltu.Atl.01_2G016610.1 ----------------------AGPG---TSDVLVVVE----------NDIK-------------------------------------------KTAQRVCEKAKQLCEAKGVS--------------TGV--EFDVAEG---DARN--   600
Soltu.Atl.03_4G017260.1 ----------------------YIPS---PAGKLSSSQLTREQVQAYINEES-------------------------------------------NRRKNLLEKYIRLCNDAKV----------------PV--DTVLVES--KSPGK--   600
Soltu.Atl.12_2G015330.1 ----------------------FEDM---DSIFASPDDFKGMKNRDKIRGLH------------------------------------------------LLEYFVNRCHEIGV----------------PC--EAWIKKG---DPKE--   600
Soltu.Atl.01_2G015420.1 ----------------------WEAT---GSPLIPLSEICDGHTMKRYGV---------------------------------------------NPDPETLQMITLAARQMEIT----------------V--VMKIFWG---DARE--   600
Soltu.Atl.08_3G009290.1 TAQQKATELQRWKAEEQRRIEEARFA---EEAALALAEKEKAKSRAALEHAEAAQRLAELESQKRINAEMKALKEAEERNKVLNKLSNSDFRYRRYSIEEIESATNYFAQSNKIGEGGYGPVYKCYMDHTHV--AVKVLRPDAAHGRQ--   600
Soltu.Atl.07_3G013530.1 ----------------------WGNT---STSPAHKIERDYEI----------------------------------------------------FTNNKATELALPLVEANVPF-------------------KIHIVKD--RDMKE--   600
Soltu.Atl.03_1G020670.1 ----------------------YIPS---PAGKLSSSQLTREQVQAYINEES-------------------------------------------NRRKNLLEKYIRLCNDAKV----------------PV--DTVLVES--KSPGK--   600
Soltu.Atl.09_3G021400.1 TSPSDSSPFSTFFR--------KSLT---NTTSPQPTSAGSHEAAEDGGDVK-----GCTG----------------------------------KGYMNFLGTMKHACEVAQPK--------------IKVCTETVEMDGNAKEKAT--   600
Soltu.Atl.05_4G020030.1 -------------------------AGPGTSDMFTLVETDIT-----------------------------------------------------KAAQKTIDKSKELCKTKGVS---------------NV--ACEILEG---DARN--   600
Soltu.Atl.06_1G017540.1 ----------------------AGPG---GADVLPYVEADLR-----------------------------------------------------KISSRIAEKAKKICSAKSVN---------------DV--VVEIVEG---DARS--   600
Soltu.Atl.06_4G012580.1 --RRQAREIDRSTSKEACNLEEAKEA---PEASHAMLEKEKQKCKAALEVAQMAQHMAELESKKRKGVEKKFRHEAEEKKKAQDAFAGSENCYRIYSTDEIEAATNYFSNSQKIGEGGYGPVYQGYLDHTSV--AIKVLRSDITQGQI--   600
Soltu.Atl.09_2G001180.2 ----------------------WAQS---GSPLIPLAEFREPEVLEKYGV---------------------------------------------EIDIELLDMLDTASRQKEIKA------------------VTKLYWG---DARE--   600
Soltu.Atl.10_4G004260.1 ----------------------WSKT---GSPLIPWVEFRDPEVMKY-YDIK-------------------------------------------KTDVQVVDLLDAATKQKEIN----------------I--VAKVYWG---DARE--   600
Soltu.Atl.01_3G023550.1 ----------------------TGEK---DHPEGYLFSSDILATMERYSS---------------------------------------------EVAQCVMEKAKRACEALNGV---------------KV--ETIVEHG---DARD--   600
Soltu.Atl.01_3G032150.1 -----------------------------------VSSLKNEIVYEATQGL--------------------------------------------IEKLAVEAFSVAMVKT-----------------------VARIVEG---DAGK--   600
                        .......460.......470.......480.......490.......500.......510.......520.......530.......540.......550.......560.......570.......580.......590.......600
```

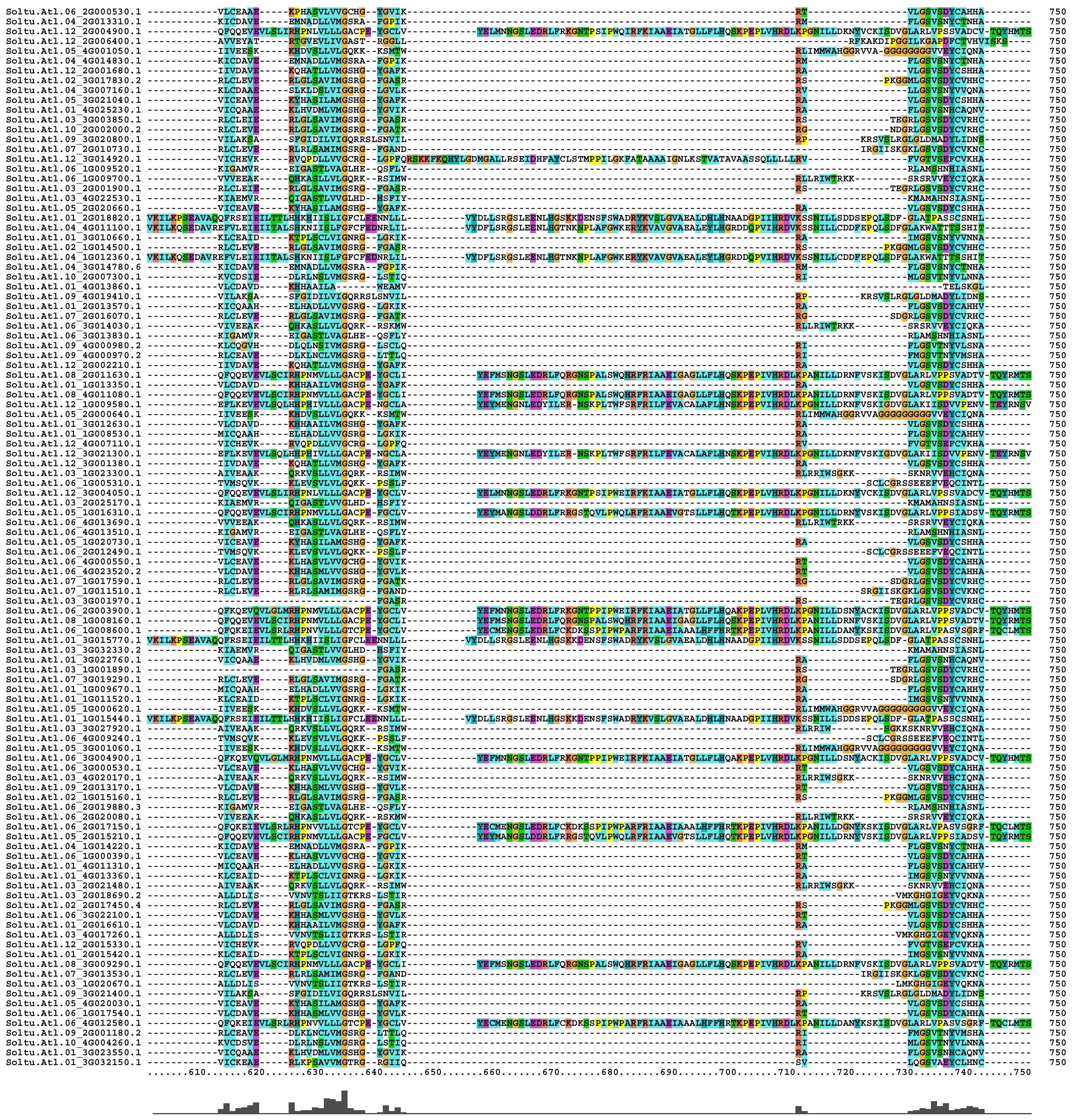

Supplement: Supplementary file 1 [file ijms-25-01341-s001.zip › ijms-2794988-supplementary/Figure S1 Multiple sequence alignment of StUSP.pdf]
